# Effects and Mechanisms of Symbiotic Microbial Combination Agents to Control Tomato *Fusarium* Crown and Root Rot Disease

**DOI:** 10.3389/fmicb.2021.629793

**Published:** 2021-06-17

**Authors:** Xinyue Cai, Honghai Zhao, Chen Liang, Min Li, Runjin Liu

**Affiliations:** ^1^Institute of Mycorrhizal Biotechnology, Qingdao Agricultural University, Qingdao, China; ^2^Key Laboratory of Integrated Crop Pest Management of Shandong Province, College of Plant Health and Medicine, Qingdao Agricultural University, Qingdao, China

**Keywords:** arbuscular mycorrhizal fungi, plant growth-promoting rhizobacteria, *Trichoderma* spp., combined microbe agents, tomato, *Fusarium* crown and root rot

## Abstract

This study evaluated the effects and underlying mechanisms of different combinations of plant symbiotic microbes, comprising arbuscular mycorrhizal fungi (AMF), plant growth-promoting rhizobacteria (PGPR), and *Trichoderma* spp., on tomato *Fusarium* crown and root rot (TFCRR) resistance. A total of 54 treatments were applied in a greenhouse pot experiment to tomato (*Solanum lycopersicum*) seedlings inoculated with or without *Funneliformis mosseae* (Fm), *Rhizophagus intraradices* (Ri), *Trichoderma virens* l40012 (Tv), *Trichoderma harzianum* l40015 (Th), *Bacillus subtilis* PS1-3 (Bs), *Pseudomonas fluorescens* PS2-6 (Pf), and *Fusarium oxysporum* f. sp. *radicis-lycopersici* (Fo). The symbioses on the tomato root system were well developed, and the composite symbiont generated by AMF + *Trichoderma* spp. was observed for the first time. Compared with other treatments, Ri + Bs + Tv and Fm + Pf + Tv stimulated the greatest improvements in tomato growth and yield. The combination Ri + Pf + Th + Fo resulted in the strongest biocontrol effects on TFCRR, followed by the treatments Th + Pf + Fo and Ri + Th + Fo. Compared with the Fo treatment, most inoculation treatments improved photosynthetic performance and significantly increased defense enzyme activity in tomato plants, of which the treatment Ri + Pf + Th + Fo showed the highest enzyme activity. Metabolome analysis detected changes in a total of 1,266 metabolites. The number of up-regulated metabolites in tomato plants inoculated with Ri + Pf + Th and Ri + Pf + Th + Fo exceeded that of the Fo treatment, whereas the number of down-regulated metabolites showed the opposite trend. It is concluded that AMF + *Trichoderma* + PGPR is the most effective combination to promote resistance to TFCRR in tomato. The up-regulation and down-regulation of metabolites regulated by symbiotic microbial genes may be an important mechanism by which root symbiotic microorganisms promote plant growth, increase yield, and improve disease resistance.

## Introduction

With the increase in prevalence of multiple cropping regimes and aggravation of obstacles to continuous cropping, soil-borne diseases increasingly restrict tomato (*Solanum lycopersicum*) production, especially under protected cultivation. Among such diseases, tomato *Fusarium* crown and root rot (TFCRR) has become a highly destructive soil-borne disease (Kucharek et al., 2000; Hibar et al., 2006; Cao et al., 2018; Li et al., 2018). Tomato production is impacted by TFCRR in more than 10 provinces and cities in China (Li et al., 2018; Wang et al., 2019).

A variety of chemical, cultivation, breeding, microbial, and management measures have been attempted to control TFCRR. At present, fungicides are mainly employed in tomato production to control soil-borne diseases. However, fungicides pollute the environment and directly contribute to toxic residues in food products, thereby endangering human health. Development of green technologies for disease prevention and control is a matter of urgency. Plant metabolites and plant-based derivative treatments have been recently used to reduce incidence of TFCRR and could replace certain chemical fungicides, such as hymexazol and benomyl (Hibar et al., 2007; Murugesan et al., 2011). Selection of resistant cultivars and grafting onto disease-resistant rootstock are effective means of prevention (Thorpe and Jarvis, 1981). However, these methods and approaches have certain limitations. Therefore, it is necessary to develop more efficient and time-saving preventive and control technologies.

Plant roots are colonized with a variety of symbiotic microorganisms, such as mycorrhizal fungi, dark septate endophytes (DSE), *Trichoderma* spp., *Metarhizium* spp., and plant growth-promoting rhizobacteria (PGPR). Each of these plant-symbiotic microorganisms may directly or indirectly inhibit infection by pathogens and promote growth of the host plant (Liu and Wang, 2018; Atalla et al., 2020; Mendes et al., 2020; Rana et al., 2020). Although some biological control agents can effectively control TFCRR in a greenhouse, the effect is more efficient when used in combination with other control measures (Colak and Bicici, 2013). Under a field environment, dual inoculation with arbuscular mycorrhizal fungi (AMF) and *Bacillus* sp. reduces fertilizer application by 50% of the recommended NPK fertilization without compromising crop growth, nutrition, and yield (Nanjundappa et al., 2019). Combined inoculation with *Funneliformis mosseae* + *Bacillus* sp. M3-4, or with *Glomus versiforme*+ *Bacillus* sp. M3-4 promotes the growth of potato and induces an enhanced defense response to control bacterial wilt disease compared with the benefits of single inoculations (Tan et al., 2015). Liu et al. (2016) evaluated the effects of different combinations of the AMF *F. mosseae* (Fm), *Rhizophagus intraradices* (Ri), and *G. versiforme* (Gv) with PGPR PR2-1, PS1-3, PS1-5, PS2-6, and PS3-2 on plant growth, resistance to *Fusarium* wilt, and yield of cucumber in a greenhouse pot experiment. The authors concluded that the combinations Fm + PS1-5, Fm + PS3-2, and Gv + PS2-6 were the most effective for control of *Fusarium* wilt among all tested treatments.

Mutualisms of plant + fungi, plant + bacteria, and fungi + bacteria often represent a reciprocal symbiotic system and form a compound symbiont in a natural ecosystem. Combined symbionts may exert more powerful functions than when inoculated alone. Field experiments show that AMF + *Trichoderma harzianum* effectively reduces the incidence of soil-borne disease and improves the quality of *Salvia miltiorrhiza* (Wang et al., 2014). Liu et al. (2019) observed improved control of tomato root knot nematode by the combined inoculation of *T. harzianum* + *Bacillus cereus* compared with that of the single strain in a field trial. *T. harzianum* mainly inhibits *Rhizoctonia solani* by reparasitism, and *Bacillus subtilis* inhibits *R. solani* by an antagonistic mechanism. The combination of these disparate inhibitory mechanisms effectively prevents potato black scurf by *R. solani* (Pan et al., 2020).

Symbiotic microbial agents in combination may exert their effects by regulating plant physiological metabolism. Inoculation of *Fragaria* × *ananassa* plants with the mycorrhizal preparations Mykoflor (*Rhizophagus irregularis*, *F. mosseae*, and *Claroideoglomus etunicatum*), MYC 800 (*R. intraradices*), and the bacterial preparation Rhizocell C (*Bacillus amyloliquefaciens* IT45) increases the transpiration rate and CO_2_ concentration in the intercellular spaces of the leaves, and the total number of bacteria and fungi in the soil is increased. Plants treated with MYC 800 + Rhizocell C show a higher CO_2_ assimilation rate than that of the control (Mikiciuk et al., 2019). The combination of AMF + PGPR reduces cucumber *Fusarium* wilt disease incidence and improves the disease resistance of cucumber plants by antagonizing pathogens, promoting synthesis of disease resistance signaling substances, up-regulating defense gene expression, increasing defense enzyme activity, and reducing accumulation of toxic substances in plants (Liu et al., 2017).

Although many previous studies have investigated TFCRR and its prevention, control of soil-borne diseases with chemical pesticides is difficult under a continuous cropping system. Therefore, development of green technologies for prevention and control of soil-borne diseases has attracted considerable research attention. As a symbiotic and active region of plant nutrient acquisition and strong interactions among many organisms, the rhizosphere is an important environment for the development of soil-borne diseases and is the principal location of plant–soil–microorganism interactions. A large number of symbiotic microbes colonize the root zone, restrict and promote each other, and exert diverse physiological and ecological effects. Therefore, it is of theoretical and practical importance to explore the effects and mechanisms of combinations of microbial agents composed of a variety of symbiotic microorganisms. Previously identified biocontrol agents were mostly single strains, but recent studies have revealed that many microbial agents show greater application potential in combination. These results lay a sound theoretical and practical foundation for further research on combination of symbiotic microbial agents. As important members of the plant root symbiotic microbiota, AMF, PGPR, and *Trichoderma* interact with each other and have been extensively studied. Compared with AMF + PGPR, inoculation with AMF + *Trichoderma*, *Trichoderma* + PGPR, and other combinations have been studied less intensively, and even fewer studies have investigated AMF + PGPR + *Trichoderma* combinations.

Metabolomic profiling is an increasingly important technique in the large field of systems biology (Dearth et al., 2018). Yang et al. (2020) used liquid chromatography combined with time-of-flight mass spectrometry (LC/TOF-MS) to analyze the metabolite profile of *Puccinellia tenuiflora* seedlings inoculated with or without AMF under alkali stress. Their findings provide insight into the metabolic mechanisms of *P. tenuiflora* seedlings infected with AMF in alkaline soil and clarify the role of AMF in the molecular regulation of this species under alkali stress. Lu et al. (2020) used ultra-performance liquid chromatography (UPLC) coupled with tandem mass spectrometry (MS/MS) to show that *F. mosseae* alleviates root rot caused by continuous cropping. The increased activity of certain disease-resistant metabolites may partly account for the disease resistance of the plants. The study provides insight into the molecular mechanism by which AMF alleviates root rot of soybean. Hill et al. (2018) performed the first metabolomic investigation into the impact of AMF colonization by *R. irregularis* on the chemical defenses in above- and belowground tissues of ragwort (*Senecio jacobaea*). An increase in the concentrations of four pyrrolizidine alkaloids in roots (but not shoots) of AMF-colonized plants was observed, which may protect colonized plants from attack by belowground organisms.

The objective of the present study was to evaluate the effect of inoculation with AMF, *Trichoderma* spp., and PGPR, either alone or in combinations, on resistance to TFCRR and to explore the possible mechanisms of the biocontrol effects.

## Materials and Methods

### Experimental Materials

Seeds of tomato ‘Ginobili’ (a cultivar sensitive to TFCRR) were purchased from Qingdao Wufeng Fruit and Vegetable Development Co. Ltd. (Qingdao, China).

The symbiotic AMF *F. mosseae* (Fm), *R. intraradices* (Ri), PGPR *B. subtilis* PS1-3 (Bs), and *P. fluorescens* PS2-6 (Pf) were obtained from the Institute of Mycorrhizal Biotechnology, Qingdao Agricultural University (QAU), China. Spores, mycorrhizal root segments, and culture media of AMF were employed as inocula with inoculation potential units determined following the method of Liu and Luo (1994).

The aforementioned PGPR species were inoculated into nutrient broth (3 g beef extract, 10 g peptone, 5 g NaCl, and distilled water to 1,000 ml) and cultured on a rotating shaker at 30°C for 3 days. The PGPR fermentation broth was diluted to 1 × 10^8^ CFU/ml for inoculation.

*Trichoderma virens* 140012 (Tv), *T. harzianum* 140015 (Th), and the TFCRR pathogen *F. oxysporum* f. sp. *radicis-lycopersici* (Fo) were provided by the College of Plant Medicine, QAU. The *Trichoderma* spp. were inoculated onto potato dextrose agar (PDA) medium and incubated at 28°C for 10 days. The pellets were filtered, washed, resuspended, and diluted to 1 × 10^5^ CFU/ml for inoculation. Fo was cultured on PDA plates for 5–7 days and a conidia suspension was prepared. The solution was diluted to 10^7^ conidia/ml, stored at 4°C, and inoculated within 24 h.

Nutrient broth and PDA were used for isolation and culture of bacteria and fungi, respectively.

Peat (0–6 mm grade; Pindstrup Mosebrug A/S, Ryomgård, Denmark) was sterilized (121°C for 1 h). The chemical characteristics of the peat were pH 6, electrical conductivity 40 mS/m, NO_3_^–^-N 70 g/m^3^, NH_4_^+^-N 50 g/m^3^, P_2_O_5_ 140 g/m^3^, K_2_O 240 g/m^3^, MgO 23 g/m^3^, B 0.4 g/m^3^, Mo 2.04 g/m^3^, Cu 1.7 g/m^3^, Mn 2.9 g/m^3^, Zn 0.9 g/m^3^, and Fe 8.4 g/m^3^. The 2.5-L pots were surface-sterilized before filling with the peat.

### Experimental Design, Inoculation, and Management

A greenhouse pot experiment was conducted with tomato seedlings inoculated with (alone or in combination) the symbiotic microbes and the pathogen, and a total of 54 treatments were designed (Supplementary Table 1). A complete randomized block design was used with five replicates for each treatment. The 54 treatments comprised inoculation with or without AMF, PGPR, and *Trichoderma*, respectively. First, 50 g of AMF inoculum (12,000 inoculation potential units), 10 ml (1 × 10^8^ CFU/ml) of each PGPR species inoculum, and/or 10 ml (1 × 10^5^ CFU/ml) of *Trichoderma* inoculum were mixed into the peat for the inoculation treatments, respectively. The same volume of sterilized inoculum was added for the non-inoculation treatment (control). Five seeds were sown in each pot. Subsequently, three seedlings were retained in each pot. At the 2–3 leaves stage, the seedlings were inoculated by irrigation with 10 ml of Fo conidial suspension applied to the root zone. Pots were watered three times weekly and, once per week, plants were fertigated with Hoagland’s nutrient solution. The volume of nutrient solution applied to each seedling in one application was 250 ml. During the growing period, the average day/night temperatures were 19°C/27°C.

### Determination of AMF Colonization, Physiological Parameters, and Metabolome Analysis of Tomato Plants

#### AMF Colonization and Associated Indices

Colonization by AMF was determined following the method described by Biermann and Linderman (1981). For PGPR population measurement, 1.0 g dry soil collected from the rhizosphere was added to sterilized water to prepare a soil solution. The PGPR numbers in the rhizosphere soil were counted using a dilution plate method after incubation at 30°C for 3 days (Bashan et al., 1993; Zhao and He, 2002). For *Trichoderma* colonization measurement, 1 g fine roots were cleaned and surface sterilized, cut into small segments and slices, and placed on PDA for culture at 25°C for 7 days. The number of effective colonies was recorded daily. The disease grade was classified as follows: grade 0, the root showed normal color; grade 1, root slightly discolored with the discolored area as much as 25% of the total root area; grade 2, roots with 26–50% of the total area showing discoloration; grade 3, 51–75% of the total root area showing discoloration; grade 4, more than 76% of the total root area is discolored or the plant is dead. The aforementioned parameters were measured at harvest.

#### Measurements of Plant Growth Indices and Photosynthetic Characteristics

Conventional methods were used to measure plant height, dry mass weight, fruit yield, and fruit weight per plant. Leaf photosynthetic parameters were measured using a portable photosynthesis system (LI-6400; LI-COR, Inc., Lincoln, NE, United States) between 08:00 and 11:30. Leaf gas exchange measurements were conducted on three leaves. The sampled leaves were frozen in liquid nitrogen and stored at −80°C for subsequent measurements of antioxidant enzyme activities.

#### Antioxidant Enzyme Activities

Fresh leaf samples (0.5 g) from tomato plants at the fruit-setting stage were homogenized with a pestle in an ice-cold mortar containing 10 ml of ice-cold 50 mM phosphate buffer (pH 7.0). The homogenate was filtered through gauze and then centrifuged at 1,500 × *g* for 20 min at 4°C. The supernatant (enzyme extract) was collected and used for the measurement of superoxide dismutase (SOD), catalase (CAT), peroxidase (POD), and phenylalanine ammonia-lyase (PAL) activities. Each sample was measured three times. The activities of SOD, CAT, POD, and PAL were determined according to the methods described by Wang (2006).

#### Metabolome Analysis

Samples of stems with young and mature leaves, and of the fine feeding roots of tomato plants at the fruit-setting stage were frozen in liquid N_2_ and stored at −80°C. Metabolome analysis was performed by Biomarker Technologies Co., Ltd. (Beijing, China) using non-target liquid chromatography–mass spectrometry (LC-MS; Biomarker Technologies) to identify differences in the metabolite profile among treatments. The specific experimental methods were as follows. The sample (50 mg) was added to 1 ml extract containing an internal standard (1000:2) (methanol/acetonitrile/water, 2:2:1, v/v/v; internal standard concentration 2 mg/L), swirled, and mixed for 30 s. Porcelain beads were added and the sample was homogenized at 45 Hz for 10 min, then treated with ultrasound for 10 min in an ice water bath. After standing for 1 h at −20°C, the homogenate was centrifuged at 4°C and 13,000 × *g* for 15 min. A sample (500 μl) of the supernatant was carefully removed from the tube. The extract was dried in a vacuum concentrator. An aliquot (150 μl) of the extract (acetonitrile/water, 1:1, v/v) was added to the dried metabolites for resolution. After vortexing for 30 s, the solution was treated with ultrasound for 10 min in an ice water bath. The solution was centrifuged at 4°C and 13,000 × *g* for 15 min. A sample (120 μl) of the supernatant was carefully transferred to a 2-ml injection flask and mixed with 10 μl from each sample of the QC sample for analysis. KEGG database was used for annotation and enrichment analysis of differential metabolites.

### Data Analysis

The data were statistically analyzed by ANOVA with DPS 7.05 software. Fisher’s protected least significant difference (LSD) was used to compare the means at 5% level of significance. The data in the table were mean ± SE.

## Results

### Root Symbionts Formed With the Symbiotic Microbes

The tested AMF, PGPR, *Trichoderma* spp., and their combinations colonized tomato roots and the rhizosphere soil. Among these microorganisms, under the condition of single inoculation and Fo inoculation, the colonization frequency of *T. virens* was significantly higher than that of *T. harzianum*, which were 1.1 and 1.2 times of that, respectively. Both Fm and Pf increased colonization by the other microorganism. Inoculation with Fm, Ri, and Pf enhanced the colonization frequency of *T. harzianum*, whereas Fo reduced colonization by AMF, PGPR, and *Trichoderma* spp. to different degrees. The AMF and *Trichoderma* sp. simultaneously colonized roots to form dual symbionts (Table 1).

**TABLE 1:** Colonization of AMF, PGPR, and *Trichoderma* spp. on tomato roots.

Treatments	AM (%)	Arbuscule (%)	PGPR (10^6^ CFU⋅g^–1^)	*Trichoderma* spp. (10^4^ CFU⋅g^–1^)
Control	–	–	–	–
Fm	84.9 ± 2.3bcd	65.5 ± 2.1ab	–	–
Ri	88.4 ± 1.1ab	44.3 ± 1.1hi	–	–
Bs	–	–	8.1 ± 0.4de	–
Pf	–	–	9.0 ± 0.4bc	–
Th	–	–	–	7.2 ± 0.3def
Tv	–	–	–	7.9 ± 0.3abc
Fm + Bs	74.6 ± 2.1hijk	56.3 ± 1.7cd	6.7 ± 0.2jkl	–
Fm + Pf	91.6 ± 2.1a	70.8 ± 2.9a	9.7 ± 0.1a	–
Ri + Bs	68.1 ± 1.5lm	48.5 ± 1.8gh	6.7 ± 0.2jkl	–
Ri + Pf	90.7 ± 1.4a	61.1 ± 1.2bc	9.0 ± 0.2b	–
Fm + Th	78.2 ± 1.3fghi	60.4 ± 1.1bc	–	8.1 ± 0.2ab
Fm + Tv	83.5 ± 1.0cde	65.8 ± 1.4ab	–	7.8 ± 0.1abc
Ri + Th	89.4 ± 1.1ab	60.4 ± 1.1bc	–	8.0 ± 0.2abc
Ri + Tv	78.1 ± 1.7ghi	50.2 ± 3.5efg	–	6.6 ± 0.4gh
Th + Bs	–	–	7.7 ± 0.2efg	6.7 ± 0.2fgh
Th + Pf	–	–	8.9 ± 0.2bc	8.2 ± 0.2a
Tv + Bs	–	–	7.7 ± 0.2efg	7.5 ± 0.3cde
Tv + Pf	–	–	8.9 ± 0.2bc	7.8 ± 0.1abcd
Fm + Bs + Th	67.1 ± 2.9m	40.9 ± 2.4ijk	5.8 ± 0.1mno	4.4 ± 0.2m
Fm + Bs + Tv	75.7 ± 1.1hij	48.2 ± 1.7gh	6.3 ± 0.1lm	5.5 ± 0.1l
Ri + Pf + Th	87.5 ± 1.2abc	59.0 ± 0.7cd	9.0 ± 0.1b	7.9 ± 0.2abc
Ri + Pf + Tv	78.0 ± 1.3ghi	44.2 ± 3.7hi	8.0 ± 0.2de	5.8 ± 0.2kl
Ri + Bs + Th	67.1 ± 2.0m	33.6 ± 1.2lmn	6.8 ± 0.1ijkl	5.7 ± 0.2l
Ri + Bs + Tv	58.0 ± 2.1n	29.8 ± 1.2mn	6.4 ± 0.2kl	5.5 ± 0.2l
Fm + Pf + Th	70.9 ± 1.3jklm	49.3 ± 4.2gh	6.5 ± 0.2kl	7.8 ± 0.2abcd
Fm + Pf + Tv	79.1 ± 1.8efgh	58.9 ± 2.7cd	7.8 ± 0.2def	8.3 ± 0.1a
Fo	–	–	–	–
Fm + Fo	70.4 ± 1.6klm	55.9 ± 2.6cde	–	–
Ri + Fo	73.5 ± 1.3ijk	40.1 ± 1.0ijk	–	–
Bs + Fo	–	–	6.5 ± 0.1kl	–
Pf + Fo	–	–	7.5 ± 0.1efgh	–
Th + Fo	–	–	–	5.9 ± 0.2jkl
Tv + Fo	–	–	–	6.8 ± 0.2fgh
Fm + Bs + Fo	59.1 ± 1.0n	38.9 ± 2.2ijkl	5.6 ± 0.3no	–
Fm + Pf + Fo	85.1 ± 2.7bcd	68.3 ± 1.0a	6.8 ± 0.1ijkl	–
Ri + Bs + Fo	57.1 ± 2.2n	28.3 ± 1.4n	5.4 ± 0.2o	–
Ri + Pf + Fo	84.5 ± 2.2bcd	53.2 ± 1.0defg	8.4 ± 0.2cd	–
Fm + Th + Fo	70.7 ± 0.8klm	49.9 ± 1.2fgh	–	5.7 ± 0.2kl
Fm + Tv + Fo	78.1 ± 1.5ghi	56.5 ± 1.9cd	–	6.6 ± 0.2ghi
Ri + Th + Fo	81.1 ± 2.3defg	55.8 ± 3.2cde	–	6.4 ± 0.1hij
Ri + Tv + Fo	68.1 ± 1.5lm	35.2 ± 2.1klm	–	5.7 ± 0.2kl
Th + Bs + Fo	–	–	6.9 ± 0.2hijk	5.8 ± 0.2kl
Th + Pf + Fo	–	–	8.9 ± 0.1bc	7.5 ± 0.2bcde
Tv + Bs + Fo	–	–	6.7 ± 0.2jkl	6.3 ± 0.1hijk
Tv + Pf + Fo	–	–	7.7 ± 0.2efg	7.1 ± 0.2efg
Fm + Bs + Th + Fo	48.8 ± 1.9o	30.4 ± 1.8mn	5.8 ± 0.3mno	4.8 ± 0.2m
Fm + Bs + Tv + Fo	60.6 ± 1.2n	41.4 ± 1.1ij	5.8 ± 0.2mno	5.4 ± 0.2l
Ri + Pf + Th + Fo	83.0 ± 1.6cdef	55.4 ± 2.7cdef	9.0 ± 0.1bc	7.5 ± 0.2bcde
Ri + Pf + Tv + Fo	72.0 ± 1.8jkl	36.4 ± 1.8jkl	7.1 ± 0.4ghij	6.8 ± 0.1fgh
Ri + Bs + Th + Fo	57.1 ± 2.0n	28.0 ± 1.3n	6.2 ± 0.2lmn	5.5 ± 0.3l
Ri + Bs + Tv + Fo	61.0 ± 1.5n	29.6 ± 1.3mn	5.7 ± 0.2mno	5.8 ± 0.2jkl
Fm + Pf + Th + Fo	58.9 ± 0.7n	38.1 ± 1.5jkl	7.4 ± 0.1fghi	6.8 ± 0.2fgh
Fm + Pf + Tv + Fo	66.6 ± 2.4m	48.2 ± 2.0gh	6.6 ± 0.4jkl	6.0 ± 0.1ijkl

*Fm, Funneliformis mosseae; Ri, Rhizophagus intraradices; Bs, Bacillus subtilis PS1-3; Pf, Pseudomonas fluorescens PS2-6; Tv, Trichoderma virens 140012; Th, Trichoderma harzianum 140015; Fo, Fusarium oxysporum f. sp. radicis-lycopersici. Mean data ± SE of 54 treatments are presented. For each column, data followed by the same letter are not significantly different at p < 0.05 (LSD test).*

### Effects of Symbiotic Microbes on Plant Growth, Development, and TFCRR

Inoculation with Fm, Ri, Bs, Pf, Th, Tv, and their combination treatments increased the growth of tomato plants, and promoted early flowering, percentage fruit set, number of fruit, and yield per plant to various degrees. The treatments Ri + Bs + Tv and Fm + Pf + Tv showed the most highly significant effects. Under the condition of Fo inoculation, the effect of the treatment Ri + Pf + Th was greatest (Table 2).

**TABLE 2:** Influences of AMF, PGPR, *Trichoderma* spp., and *Fusarium oxysporum* f. sp. *radicis-lycopersici* on tomato growth and yield.

Inoculation	Plant height (cm)	Dry mass of stem with leaves (g)	Dry mass of roots (g)	Fruit weight per plant (g)	Number of fruits per plant
Control	42.2 ± 4.3gh	26.7 ± 1.3u	4.2 ± 0.2opqrst	20.5 ± 6.4lm	1.7 ± 0.3de
Fm	46.3 ± 1.9cdefgh	35.2 ± 1.1pqrs	4.6 ± 0.2mnopqr	35.7 ± 0.6fghijkl	1.7 ± 0.3de
Ri	45.6 ± 3.2cdefgh	36.5 ± 1.8lmnopqr	4.5 ± 0.4mnopqrs	66.6 ± 15abc	2.0 ± 0.6cde
Bs	50.5 ± 4.5abcdefgh	42.5 ± 1.9cdefghij	6.2 ± 0.2ab	31.6 ± 3.2hijklm	2.0 ± 0.0cde
Pf	46.9 ± 2.6bcdefgh	34.4 ± 3.0qrst	4.5 ± 0.2mnopqrs	36.4 ± 3.6efghijkl	2.0 ± 0.0cde
Th	53.2 ± 2.6abcdef	44.0 ± 1.7bcdef	6.8 ± 0.3a	28.0 ± 8.0hijklm	2.0 ± 0.6cde
Tv	46.3 ± 3.8cdefgh	36.4 ± 3.0lmnopqr	5.3 ± 0.3efghijkl	68.5 ± 4.2ab	2.0 ± 0.0cde
Fm + Bs	55.8 ± 3.9abc	47.6 ± 2.9bcd	6.0 ± 0.1bc	18.9 ± 3.2lm	1.7 ± 0.3de
Fm + Pf	50.2 ± 4.0abcdefgh	45.4 ± 3.0bcdef	5.3 ± 0.3efghijkl	48.8 ± 4.4bcdefghij	2.3 ± 0.3bcde
Ri + Bs	49.9 ± 4.5bcdefgh	43.3 ± 2.2bcdefghij	5.3 ± 0.3defghijk	63.5 ± 8.5abcd	2.3 ± 0.3bcde
Ri + Pf	51.5 ± 4.5abcdefgh	46.3 ± 2.2bcdef	5.6 ± 0.3cdefgh	50.8 ± 5.7bcdefgh	3.0 ± 0.0abcd
Fm + Th	54.4 ± 1.8abcd	48.4 ± 1.8bc	5.9 ± 0.2bcde	26.6 ± 8.5ijklm	3.0 ± 0.0abcd
Fm + Tv	49.3 ± 5.0bcdefgh	44.1 ± 1.2bcdef	5.0 ± 0.1ghijklmn	41.1 ± 8.4defghijkl	2.7 ± 0.3bcde
Ri + Th	46.6 ± 3.6bcdefgh	36.3 ± 3.0lmnopqr	4.5 ± 0.3mnopqrs	36.4 ± 5.9efghijkl	1.7 ± 0.7de
Ri + Tv	47.4 ± 3.5bcdefgh	37.7 ± 2.2ghijklmnopq	4.7 ± 0.2klmnopq	55.4 ± 6.3abcdefg	2.0 ± 0.0cde
Th + Bs	50.5 ± 7.9abcdefgh	43.6 ± 2.0bcdefg	5.7 ± 0.3bcdef	40.5 ± 6.6defghijkl	2.7 ± 1.2bcde
Th + Pf	51.4 ± 2.2abcdefgh	46.6 ± 3.1bcde	5.9 ± 0.2bcde	47.5 ± 7.3bcdefghijk	2.3 ± 0.3bcde
Tv + Bs	50.3 ± 3.9abcdefgh	42.6 ± 2.4cdefghij	5.4 ± 0.3defghijk	59.9 ± 14abcde	2.3 ± 0.3bcde
Tv + Pf	49.5 ± 3.6bcdefgh	41.2 ± 2.3efghijklmno	4.7 ± 0.2klmnopq	66.6 ± 9.4abc	2.7 ± 0.3bcde
Fm + Bs + Th	47.7 ± 3.9bcdefgh	36.5 ± 1.8klmnopqr	4.3 ± 0.2nopqrst	36.4 ± 5.4efghijkl	3.0 ± 0.6abcd
Fm + Bs + Tv	51.6 ± 3.5abcdefgh	46.4 ± 1.3bcde	5.7 ± 0.3bcdef	45.6 ± 4.8bcdefghijk	2.3 ± 0.3bcde
Ri + Pf + Th	43.4 ± 2.8fgh	34.7 ± 1.8pqrs	4.4 ± 0.2nopqrst	37.0 ± 8.0efghijkl	2.0 ± 1.0cde
Ri + Pf + Tv	45.2 ± 3.5defgh	35.6 ± 1.1nopqr	4.6 ± 0.2lmnopqr	41.8 ± 6.0defghijkl	2.7 ± 0.3bcde
Ri + Bs + Th	53.5 ± 2.4abcdef	45.3 ± 3.0bcdef	5.6 ± 0.3bcdefg	36.5 ± 3.1efghijkl	2.3 ± 0.9bcde
Ri + Bs + Tv	60.5 ± 1.4a	57.5 ± 0.7a	6.0 ± 0.3bcd	50.1 ± 2.2bcdefghi	3.3 ± 0.3abc
Fm + Pf + Th	48.6 ± 4.2bcdefgh	42.8 ± 2.3bcdefghij	5.5 ± 0.2cdefghi	32.7 ± 4.9ghijkl	2.0 ± 0.0cde
Fm + Pf + Tv	56.7 ± 1.6ab	54.7 ± 0.8a	5.4 ± 0.1cdefghij	76.7 ± 8.1a	3.0 ± 0.6abcd
Fo	42.4 ± 4.1gh	28.6 ± 1.3tu	4.8 ± 0.2jklmnop	29.1 ± 8.6hijklm	2.7 ± 0.7bcde
Fm + Fo	43.3 ± 4.0fgh	33.5 ± 1.8qrst	3.2 ± 0.1w	29.1 ± 13hijklm	2.0 ± 0.0cde
Ri + Fo	49.8 ± 4.6bcdefgh	42.4 ± 1.8cdefghijk	4.1 ± 0.2qrst	24.9 ± 7.2klm	1.7 ± 0.3de
Bs + Fo	47.3 ± 4.7bcdefgh	40.4 ± 2.3fghijklmnop	4.3 ± 0.1opqrst	38.8 ± 12efghijkl	2.0 ± 0.6cde
Pf + Fo	44.5 ± 4.8defgh	36.2 ± 1.8lmnopqr	4.2 ± 0.1pqrst	27.2 ± 4.4ijklm	2.0 ± 0.0cde
Th + Fo	43.6 ± 4.0efgh	35.7 ± 1.3mnopqr	3.8 ± 0.2tuv	32.4 ± 3.1ghijklm	2.0 ± 0.0cde
Tv + Fo	41.7 ± 2.2h	29.4 ± 1.2stu	3.5 ± 0.2uvw	27.8 ± 1.9hijklm	2.0 ± 0.6cde
Fm + Bs + Fo	47.4 ± 3.4bcdefgh	41.5 ± 1.7efghijklmn	4.0 ± 0.2rstu	37.8 ± 4.9efghijkl	1.7 ± 0.3de
Fm + Pf + Fo	49.7 ± 4.1bcdefgh	41.6 ± 2.1efghijklm	3.9 ± 0.1tuv	57.9 ± 16abcdef	1.7 ± 0.3de
Ri + Bs + Fo	50.1 ± 1.2bcdefgh	43.6 ± 2.0bcdefgh	4.5 ± 0.2nopqrst	25.4 ± 2.8jklm	1.7 ± 0.3de
Ri + Pf + Fo	49.4 ± 4.0bcdefgh	42.4 ± 2.2cdefghijk	4.4 ± 0.2nopqrst	39.5 ± 3.4efghijkl	2.0 ± 0.6cde
Fm + Th + Fo	44.6 ± 4.5defgh	37.6 ± 1.2hijklmnopq	3.4 ± 0.2vw	31.9 ± 5.3ghijklm	2.0 ± 0.0cde
Fm + Tv + Fo	41.5 ± 2.1h	31.2 ± 1.2rstu	3.0 ± 0.1w	38.4 ± 14efghijkl	1.3 ± 0.3e
Ri + Th + Fo	45.2 ± 3.5defgh	37.7 ± 1.6ghijklmnopq	3.9 ± 0.2stuv	49.0 ± 5.1bcdefghij	2.0 ± 0.0cde
Ri + Tv + Fo	49.8 ± 4.6bcdefgh	41.7 ± 2.4efghijkl	4.3 ± 0.1nopqrst	35.2 ± 17fghijkl	3.0 ± 1.0abcd
Th + Bs + Fo	47.7 ± 3.6bcdefgh	48.7 ± 3.3b	5.2 ± 0.3efghijkl	37.1 ± 6.2efghijkl	2.0 ± 0.6cde
Th + Pf + Fo	45.6 ± 3.8cdefgh	37.6 ± 1.2ijklmnopq	4.8 ± 0.1klmnopq	30.1 ± 9.2hijklm	1.7 ± 0.3de
Tv + Bs + Fo	52.4 ± 4.5abcdefg	44.6 ± 2.5bcdef	5.8 ± 0.2bcde	32.3 ± 6.5ghijklm	2.0 ± 0.6cde
Tv + Pf + Fo	43.5 ± 4.5efgh	35.4 ± 2.2opqr	4.8 ± 0.2ijklmno	19.5 ± 3.7lm	1.7 ± 0.3de
Fm + Bs + Th + Fo	45.8 ± 3.4cdefgh	37.5 ± 1.3jklmnopq	4.3 ± 0.2opqrst	9.1 ± 0.5m	2.0 ± 0.0cde
Fm + Bs + Tv + Fo	50.5 ± 3.3abcdefgh	43.5 ± 2.3bcdefghi	5.2 ± 0.1fghijklm	44.6 ± 12cdefghijk	2.0 ± 0.0cde
Ri + Pf + Th + Fo	53.8 ± 2.4abcde	45.5 ± 2.7bcdef	4.3 ± 0.1opqrst	49.2 ± 22bcdefghi	4.3 ± 2.3a
Ri + Pf + Tv + Fo	48.6 ± 3.4bcdefgh	42.5 ± 2.9cdefghij	4.4 ± 0.1nopqrst	37.8 ± 4.2efghijkl	2.0 ± 0.0cde
Ri + Bs + Th + Fo	47.7 ± 3.6bcdefgh	41.7 ± 2.4defghijkl	4.2 ± 0.3opqrst	38.8 ± 15efghijkl	3.7 ± 0.9ab
Ri + Bs + Tv + Fo	51.4 ± 4.0abcdefgh	44.6 ± 2.1bcdef	4.6 ± 0.3mnopqr	38.2 ± 4.0efghijkl	3.0 ± 0.6abcd
Fm + Pf + Th + Fo	49.6 ± 4.6bcdefgh	42.5 ± 2.3cdefghij	4.1 ± 0.2rstu	40.1 ± 5.8defghijkl	2.7 ± 0.3bcde
Fm + Pf + Tv + Fo	49.6 ± 4.2bcdefgh	43.5 ± 2.9bcdefghi	4.9 ± 0.3hijklmn	39.1 ± 5.5efghijkl	2.7 ± 0.3bcde

*Fm, Funneliformis mosseae; Ri, Rhizophagus intraradices; Bs, Bacillus subtilis PS1-3; Pf, Pseudomonas fluorescens PS2-6; Tv, Trichoderma virens 140012; Th, Trichoderma harzianum 140015; Fo, Fusarium oxysporum f. sp. radicis-lycopersici. Mean data ± SE of 54 treatments are presented. For each column, data followed by the same letter are not significantly different at p < 0.05 (LSD test).*

Single inoculation, and the combinations of two or three symbiotic microorganisms, reduced the incidence and disease index of TFCRR, but the effects differed among treatments. The treatment Ri + Pf + Th + Fo showed the most significant effect, followed by the Th + Pf + Fo and Ri + Th + Fo treatments. Multivariate analysis of variance showed that there were significant interactions among AMF, PGPR, and *Trichoderma* spp. (Table 3).

**TABLE 3:** Effects of AMF, PGPR, and *Trichoderma* spp. on TFCRR disease.

Inoculation	Disease incidence (%)	Disease index	Relative control effects (%)
Fo	75.7 ± 1.2a	53.1 ± 1.6a	–
Fm + Fo	35.0 ± 2.4hi	34.0 ± 2.5defgh	35.9 ± 4.8efghi
Ri + Fo	33.6 ± 1.8hi	31.6 ± 1.1ghi	40.4 ± 2.1def
Bs + Fo	53.1 ± 0.8bc	41.4 ± 0.6b	22.0 ± 1.0k
Pf + Fo	49.5 ± 1.0cd	38.3 ± 1.3bcd	28.0 ± 2.5ijk
Th + Fo	41.2 ± 1.3fg	34.1 ± 2.7defgh	35.7 ± 5.1efghi
Tv + Fo	56.7 ± 2.1b	37.8 ± 2.3bcde	28.8 ± 4.3hijk
Fm + Bs + Fo	38.3 ± 1.7ghi	35.6 ± 2.3cdefg	32.9 ± 4.3fghij
Fm + Pf + Fo	33.9 ± 1.9hi	31.5 ± 1.0ghi	40.6 ± 1.8def
Ri + Bs + Fo	47.9 ± 1.3de	39.8 ± 1.7bc	25.1 ± 3.2jk
Ri + Pf + Fo	28.2 ± 1.5jk	24.1 ± 2.5jk	54.7 ± 4.8bc
Fm + Th + Fo	44.4 ± 2.5def	40.7 ± 1.0b	23.4 ± 1.9k
Fm + Tv + Fo	38.5 ± 3.3gh	34.8 ± 2.3defgh	34.5 ± 4.4efghi
Ri + Th + Fo	26.5 ± 1.9k	22.7 ± 0.7kl	57.3 ± 1.3ab
Ri + Tv + Fo	48.3 ± 1.0cde	37.7 ± 1.2bcdef	29.0 ± 2.2ghijk
Th + Bs + Fo	33.6 ± 1.9hi	27.9 ± 1.3ij	47.5 ± 2.5cd
Th + Pf + Fo	26.1 ± 1.6k	21.9 ± 1.6kl	58.7 ± 3.1ab
Tv + Bs + Fo	48.0 ± 1.3cde	39.9 ± 1.4bc	24.8 ± 2.7jk
Tv + Pf + Fo	44.0 ± 2.5ef	30.5 ± 1.1hi	42.6 ± 2.0de
Fm + Bs + Th + Fo	38.1 ± 1.4ghi	33.8 ± 1.5defgh	36.4 ± 2.9efghi
Fm + Bs + Tv + Fo	42.6 ± 1.3fg	33.5 ± 1.1efgh	37.0 ± 2.1efgh
Ri + Pf + Th + Fo	12.8 ± 1.6l	18.1 ± 1.3l	65.8 ± 2.5a
Ri + Pf + Tv + Fo	33.4 ± 1.9hi	30.4 ± 1.1hi	42.8 ± 2.1de
Ri + Bs + Th + Fo	35.2 ± 2.1hi	30.2 ± 1.9hi	43.1 ± 3.6de
Ri + Bs + Tv + Fo	41.7 ± 2.3fg	32.9 ± 0.9fgh	38.0 ± 1.6efg
Fm + Pf + Th + Fo	35.3 ± 1.6hi	23.8 ± 2.7jk	55.1 ± 5.0bc
Fm + Pf + Tv + Fo	33.3 ± 1.4ij	23.8 ± 1.1jk	55.1 ± 2.1bc
ANOVA			
F(AMF)	**	**	**
F(PGPR)	**	**	**
F(*Trichoderma*)	**	**	**
F(AMF × PGPR)	**	**	**
F(AMF × *Trichoderma*)	**	**	**
F(PGPR × *Trichoderma*)	NS	NS	NS
F(AMF × PGPR × *Trichoderma*)	**	**	**

Fm, Funneliformis mosseae; Ri, Rhizophagus intraradices; Bs, Bacillus subtilis PS1-3; Pf, Pseudomonas fluorescens PS2-6; Tv, Trichoderma virens 140012; Th, Trichoderma harzianum 140015; Fo, Fusarium oxysporum f. sp. radicis-lycopersici. Mean data ± SE of 28 treatments are presented. For each column, data followed by the same letter are not significantly different (p < 0.05). NS means no significant difference; **means extremely significant difference at p < 0.01 level.

### Effects of Symbiotic Microbes on Photosynthesis and Antioxidant Enzyme Activities in Tomato Leaves

Most of the photosynthesis parameters, such as net photosynthetic rate (*P*_*n*_), stomatal conductance (*G*_*s*_), intercellular carbon dioxide concentration (*C*_*i*_), and transpiration rate (*T*_*r*_), of tomato leaves treated with Ri, Bs, Tv + Bs, Tv + Pf, Ri + Bs + Tv, and Ri + Pf + Tv were significantly higher than those of the non-inoculated control. Compared with the Fo treatment, most of the inoculation treatments showed improved photosynthetic performance (Table 4).

**TABLE 4:** Influences of AMF, PGPR, *Trichoderma* spp., and *Fusarium oxysporum* f. sp. *radicis-lycopersici* on tomato leaf photosynthetic performance.

Inoculation	*P*_*n*_	*G*_*s*_	*C*_*i*_	*T*_*r*_
	(μmol⋅m^–2^⋅s^–1^)	(mol⋅m^–2^⋅s^–1^)	(μmol⋅mol^–1^)	(mmol⋅m^–2^⋅s^–1^)
Control	9.3 ± 0.1ij	0.21 ± 0.06ij	265.5 ± 2.9defgh	2.3 ± 0.1hijklm
Fm	12.5 ± 1.1bcdefghi	0.32 ± 0.01bcdefgh	308.9 ± 0.5abcd	3.6 ± 0.1abcdefg
Ri	13.8 ± 0.6bc	0.38 ± 0.01bcd	319.0 ± 1.8a	4.7 ± 0.2a
Bs	13.8 ± 0.8bc	0.35 ± 0.07bcdef	313.3 ± 8.7abc	4.6 ± 0.6ab
Pf	10.1 ± 0.3ghij	0.25 ± 0.07fghi	294.8 ± 15abcdefg	2.9 ± 0.2efghijklm
Th	10.2 ± 0.2fghij	0.29 ± 0.08cdefghi	314.0 ± 5.1abc	3.5 ± 1.0abcdefgh
Tv	13.5 ± 1.6bcde	0.31 ± 0.05bcdefghi	306.7 ± 2.0abcd	4.2 ± 0.4abcd
Fm + Bs	13.0 ± 1.3bcdefgh	0.30 ± 0.03cdefghi	310.4 ± 3.1abcd	4.0 ± 0.2abcde
Fm + Pf	12.8 ± 1.1bcdefgh	0.33 ± 0.06bcdef	314.8 ± 7.1ab	4.4 ± 0.5abc
Ri + Bs	12.9 ± 0.6bcdefgh	0.34 ± 0.03bcdef	314.4 ± 4.6ab	3.7 ± 0.7abcdef
Ri + Pf	10.2 ± 0.3efghij	0.34 ± 0.16bcdef	299.1 ± 31abcdef	2.8 ± 0.1fghijklm
Fm + Th	13.2 ± 1.8bcdefg	0.26 ± 0.06fghi	289.8 ± 12abcdefg	3.1 ± 0.9defghijk
Fm + Tv	13.2 ± 1.0bcdefg	0.30 ± 0.03cdefghi	307.8 ± 9abcd	4.1 ± 0.2abcde
Ri + Th	10.3 ± 3.5efghij	0.32 ± 0.01bcdefgh	299.5 ± 10abcdef	2.9 ± 1.1efghijklm
Ri + Tv	12.3 ± 0.8bcdefghi	0.35 ± 0.01bcdef	284.9 ± 3.2abcdefgh	3.2 ± 0.2cdefghijk
Th + Bs	12.5 ± 0.3bcdefghi	0.36 ± 0.01bcde	304.0 ± 1.1abcde	3.8 ± 0.1abcdef
Th + Pf	14.1 ± 1.1ab	0.27 ± 0.05efghi	288.9 ± 9.3abcdefg	3.7 ± 0.5abcdef
Tv + Bs	12.8 ± 0.6bcdefgh	0.41 ± 0.01ab	317.6 ± 11ab	3.6 ± 0.2abcdefg
Tv + Pf	17.3 ± 2.5a	0.37 ± 0.01bcde	294.8 ± 3.3abcdefg	4.7 ± 0.6a
Fm + Bs + Th	10.1 ± 2.7ghij	0.35 ± 0.01bcdef	306.5 ± 13abcd	3.1 ± 1.2defghijkl
Fm + Bs + Tv	12.4 ± 1.6bcdefghi	0.37 ± 0.01bcde	301.7 ± 8.7abcdef	3.2 ± 0.5cdefghijk
Ri + Pf + Th	12.3 ± 0.5bcdefghi	0.31 ± 0.01bcdefghi	188.0 ± 30j	2.9 ± 0.1efghijklm
Ri + Pf + Tv	13.0 ± 0.4bcdefgh	0.33 ± 0.01bcdefg	304.4 ± 7.3abcd	3.8 ± 0.3abcdef
Ri + Bs + Th	10.6 ± 1.4cdefghi	0.38 ± 0.01abc	276.1 ± 20abcdefgh	2.3 ± 0.8hijklm
Ri + Bs + Tv	13.6 ± 1.3bcd	0.48 ± 0.01a	291.9 ± 12abcdefg	2.3 ± 0.4ijklmn
Fm + Pf + Th	11.1 ± 2.2bcdefghi	0.30 ± 0.01cdefghi	278.3 ± 21abcdefgh	2.1 ± 0.7jklmn
Fm + Pf + Tv	12.1 ± 0.7bcdefghi	0.31 ± 0.01bcdefghi	303.4 ± 18abcdef	3.4 ± 1.1bcdefghi
Fo	7.3 ± 0.1j	0.14 ± 0.01j	212.9 ± 1.5ij	1.1 ± 0.1n
Fm + Fo	11.6 ± 0.7bcdefghi	0.33 ± 0.03bcdef	256.6 ± 29fghi	2.0 ± 0.2klmn
Ri + Fo	12.6 ± 0.8bcdefghi	0.29 ± 0.01cdefghi	248.7 ± 26ghi	1.8 ± 0.1mn
Bs + Fo	13.5 ± 1.0bcde	0.27 ± 0.01defghi	270.9 ± 32bcdefgh	2.0 ± 0.1jklmn
Pf + Fo	11.7 ± 1.4bcdefghi	0.25 ± 0.01fghi	279.4 ± 12abcdefgh	1.8 ± 0.1mn
Th + Fo	9.8 ± 0.2hij	0.27 ± 0.01efghi	288.3 ± 21abcdefg	1.9 ± 0.1lmn
Tv + Fo	10.8 ± 1.2bcdefghi	0.26 ± 0.01efghi	266.9 ± 34cdefgh	2.0 ± 0.1jklmn
Fm + Bs + Fo	13.5 ± 1.0bcdef	0.28 ± 0.05defghi	317.6 ± 14ab	2.2 ± 0.1ijklmn
Fm + Pf + Fo	12.9 ± 0.6bcdefgh	0.29 ± 0.01cdefghi	306.8 ± 5.4abcd	2.2 ± 0.1ijklmn
Ri + Bs + Fo	11.9 ± 1.7bcde	0.32 ± 0.01bcdefgh	309.9 ± 9.7abcd	2.3 ± 0.1hijklmn
Ri + Pf + Fo	12.6 ± 0.7bcdefghi	0.23 ± 0.03ghij	301.0 ± 7.2abcdef	2.3 ± 0.1hijklmn
Fm + Th + Fo	11.5 ± 0.7bcde	0.25 ± 0.01fghi	275.2 ± 16abcdefgh	2.3 ± 0.1hijklmn
Fm + Tv + Fo	12.6 ± 0.9bcdefghi	0.27 ± 0.01defghi	289.8 ± 23abcdefg	2.4 ± 0.1ghijklm
Ri + Th + Fo	11.3 ± 1.2bcdefghi	0.26 ± 0.01efghi	281.0 ± 13abcdefgh	2.3 ± 0.1hijklm
Ri + Tv + Fo	12.5 ± 0.8bcdefghi	0.22 ± 0.09hij	282.7 ± 22abcdefgh	1.8 ± 0.1mn
Th + Bs + Fo	13.0 ± 1.1bcdefgh	0.32 ± 0.02bcdefgh	308.7 ± 11abcd	3.2 ± 0.1cdefghij
Th + Pf + Fo	12.7 ± 0.4bcdefgh	0.28 ± 0.01cdefghi	312.4 ± 12abcd	2.9 ± 0.1efghijklm
Tv + Bs + Fo	11.5 ± 0.8bcdefghi	0.33 ± 0.01bcdefg	238.7 ± 22hi	2.3 ± 0.1ijklmn
Tv + Pf + Fo	10.6 ± 0.7cdefghij	0.28 ± 0.01cdefghi	256.9 ± 51efghi	1.9 ± 0.1lmn
Fm + Bs + Th + Fo	11.6 ± 0.9bcdefghi	0.26 ± 0.01efghi	276.6 ± 10abcdefgh	2.4 ± 0.1ghijklm
Fm + Bs + Tv + Fo	10.7 ± 0.8cdefghi	0.31 ± 0.01bcdefghi	317.0 ± 11ab	2.2 ± 0.1ijklmn
Ri + Pf + Th + Fo	12.4 ± 0.7bcdefghi	0.33 ± 0.02bcdef	299.5 ± 9.3abcdef	2.3 ± 0.1ijklmn
Ri + Pf + Tv + Fo	10.5 ± 0.7cdefghij	0.26 ± 0.01efghi	298.3 ± 4.3abcdef	1.9 ± 0.1lmn
Ri + Bs + Th + Fo	10.3 ± 0.6efghij	0.32 ± 0.01bcdefgh	307.8 ± 5.4abcd	2.1 ± 0.1jklmn
Ri + Bs + Tv + Fo	11.6 ± 0.5bcde	0.26 ± 0.01efghi	296.0 ± 15abcdef	2.3 ± 0.1hijklmn
Fm + Pf + Th + Fo	10.3 ± 1.7defghij	0.25 ± 0.01fghi	291.4 ± 19abcdefg	1.8 ± 0.1mn
Fm + Pf + Tv + Fo	12.1 ± 0.7bcdefghi	0.28 ± 0.01cdefghi	292.0 ± 21abcdefg	2.3 ± 0.1hijklmn

*Fm, Funneliformis mosseae; Ri, Rhizophagus intraradices; Bs, Bacillus subtilis PS1-3; Pf, Pseudomonas fluorescens PS2-6; Tv, Trichoderma virens 140012; Th, Trichoderma harzianum 140015; Fo, Fusarium oxysporum f. sp. radicis-lycopersici. Mean data ± SE of 54 treatments are presented. For each column, data followed by the same letter are not significantly different at p < 0.05 (LSD test).*

In addition, most of the inoculation treatments significantly increased the antioxidant enzyme activity of tomato plants. Among these enzymes, POD, SOD, CAT, and PAL showed the highest activity in the treatment Ri + Pf + Th + Fo, followed by Fm + Pf + Th + Fo, Fm + Pf + Tv + Fo, Ri + Bs + Tv + Fo, Ri + Th + Fo, and Th + Pf + Fo (Table 5).

**TABLE 5:** Influences of AMF, PGPR, *Trichoderma*, and *Fusarium oxysporum* f. sp. *radicis-lycopersici* on the antioxidant enzyme activity in tomato leaves.

Inoculation	POD activity	SOD activity	CAT activity	PAL activity
	(U/g⋅min)	(U/g)	(U/g⋅min)	(U/g⋅min)
Control	10.6 ± 0.8n	57.3 ± 0.9o	82.8 ± 0.6p	10.4 ± 0.7p
Fm	23.2 ± 1.1k	85.3 ± 0.5lm	117.8 ± 0.8n	18.2 ± 0.6lmn
Ri	23.8 ± 0.7k	87.0 ± 0.6kl	117.6 ± 0.6n	19.4 ± 0.3jkl
Bs	18.4 ± 1.5m	80.0 ± 1.6n	110.0 ± 0.8o	15.3 ± 0.3o
Pf	18.7 ± 1.3m	82.5 ± 1.4mn	112.1 ± 0.6o	16.8 ± 0.6mno
Th	18.5 ± 1.2m	81.9 ± 1.4n	112.1 ± 0.9o	16.4 ± 0.6no
Tv	19.9 ± 1.3lm	81.5 ± 1.2n	112.1 ± 0.8o	16.9 ± 1.1mno
Fm + Bs	23.8 ± 0.5k	86.9 ± 0.8kl	129.2 ± 0.8l	19.4 ± 0.6jkl
Fm + Pf	29.8 ± 1.1hij	88.5 ± 0.3k	136.8 ± 0.8j	23.4 ± 0.7h
Ri + Bs	23.3 ± 1.0k	85.3 ± 0.8lm	122.4 ± 0.7m	18.5 ± 0.6klm
Ri + Pf	27.5 ± 0.6j	89.2 ± 0.6k	132.0 ± 1.2k	20.3 ± 0.6jk
Fm + Th	23.8 ± 1.2k	87.2 ± 0.7kl	127.7 ± 0.6l	19.4 ± 0.6jkl
Fm + Tv	28.8 ± 0.9ij	89.5 ± 0.9k	135.2 ± 0.4j	21.2 ± 0.7ij
Ri + Th	27.6 ± 0.8j	88.7 ± 1.3k	131.7 ± 0.7k	20.2 ± 0.5jk
Ri + Tv	23.5 ± 0.4k	85.3 ± 0.7lm	131.8 ± 0.8k	18.5 ± 0.8klm
Th + Bs	21.1 ± 1.2klm	80.5 ± 0.4n	122.2 ± 0.9m	18.2 ± 0.6lmn
Th + Pf	21.5 ± 1.0kl	82.5 ± 0.9mn	123.4 ± 0.7m	19.3 ± 1.2jkl
Tv + Bs	21.5 ± 1.2kl	81.6 ± 1.5n	124.2 ± 0.5m	18.2 ± 0.7lmn
Tv + Pf	28.5 ± 0.6ij	88.7 ± 1.1k	132.0 ± 0.8k	20.4 ± 0.6jk
Fm + Bs + Th	31.0 ± 0.7ghi	97.0 ± 1.1ij	142.1 ± 0.8hi	24.4 ± 0.5h
Fm + Bs + Tv	32.1 ± 0.9fgh	97.1 ± 1.1ij	144.1 ± 0.5h	26.7 ± 0.6g
Ri + Pf + Th	37.8 ± 0.9cd	110.5 ± 1.1fg	152.1 ± 1.2g	32.7 ± 0.5de
Ri + Pf + Tv	32.1 ± 0.9fgh	102.1 ± 0.8h	144.2 ± 1.1h	26.6 ± 0.5g
Ri + Bs + Th	30.0 ± 1.1hij	95.4 ± 0.8j	139.9 ± 0.9i	21.2 ± 0.6ij
Ri + Bs + Tv	30.0 ± 1.1hij	95.7 ± 0.6j	139.9 ± 0.7i	22.6 ± 0.7hi
Fm + Pf + Th	32.1 ± 1.1fgh	98.6 ± 0.8i	144.1 ± 0.6h	27.4 ± 0.8g
Fm + Pf + Tv	33.5 ± 1.4fg	102.0 ± 0.8h	144.2 ± 0.5h	29.7 ± 0.5f
Fo	20.1 ± 1.7lm	79.8 ± 1.2n	117.3 ± 1.3n	16.7 ± 0.7mno
Fm + Fo	30.9 ± 1.1ghi	98.5 ± 0.5i	135.4 ± 0.6j	20.6 ± 0.6j
Ri + Fo	33.2 ± 0.8fg	102.2 ± 1.0h	140.0 ± 0.9i	24.2 ± 0.5h
Bs + Fo	28.6 ± 1.1ij	94.6 ± 0.8j	128.2 ± 0.5l	20.0 ± 0.5jkl
Pf + Fo	28.6 ± 0.4ij	94.7 ± 1.4j	128.5 ± 0.4l	20.6 ± 0.7j
Th + Fo	31.2 ± 0.7ghi	98.5 ± 1.4i	136.8 ± 0.6j	23.4 ± 0.6h
Tv + Fo	30.3 ± 0.8hij	97.1 ± 1.0ij	131.6 ± 0.8k	22.7 ± 0.6hi
Fm + Bs + Fo	34.7 ± 0.4ef	108.8 ± 0.9g	144.0 ± 0.7h	27.3 ± 0.6g
Fm + Pf + Fo	36.5 ± 1.3de	112.1 ± 1.5f	153.2 ± 0.8g	30.5 ± 0.9f
Ri + Bs + Fo	36.4 ± 1.1de	110.7 ± 0.9fg	142.1 ± 0.6hi	27.5 ± 0.7g
Ri + Pf + Fo	38.2 ± 1.1cd	112.4 ± 1.3f	157.6 ± 0.7f	29.8 ± 0.5f
Fm + Th + Fo	34.7 ± 0.6ef	108.7 ± 0.9g	142.1 ± 0.9hi	27.5 ± 0.7g
Fm + Tv + Fo	36.5 ± 0.8de	109.0 ± 0.7g	152.3 ± 1.2g	26.9 ± 0.6g
Ri + Th + Fo	40.4 ± 1.5bc	120.0 ± 1.4d	162.1 ± 1.1e	33.2 ± 0.5de
Ri + Tv + Fo	34.9 ± 0.6ef	108.4 ± 0.9g	153.3 ± 0.6g	24.4 ± 0.8h
Th + Bs + Fo	36.8 ± 0.8de	110.3 ± 0.8fg	157.8 ± 0.8f	27.0 ± 0.9g
Th + Pf + Fo	40.4 ± 1.4bc	119.9 ± 1.1d	161.6 ± 0.9e	32.8 ± 0.6de
Tv + Bs + Fo	36.4 ± 1.0de	112.0 ± 1.5f	143.7 ± 0.6h	27.3 ± 1.0g
Tv + Pf + Fo	36.8 ± 0.8de	112.7 ± 0.6f	157.7 ± 0.8f	30.3 ± 0.7f
Fm + Bs + Th + Fo	38.5 ± 0.4cd	115.9 ± 0.8e	164.9 ± 1.1d	31.5 ± 0.6ef
Fm + Bs + Tv + Fo	38.1 ± 0.3cd	116.4 ± 1.4e	165.1 ± 0.8d	31.5 ± 0.6ef
Ri + Pf + Th + Fo	48.5 ± 1.1a	135.4 ± 1.2a	204.8 ± 1.1a	42.7 ± 1.2a
Ri + Pf + Tv + Fo	42.9 ± 1.3b	122.6 ± 1.2cd	181.9 ± 1.0b	34.6 ± 0.6cd
Ri + Bs + Th + Fo	40.5 ± 0.6bc	122.6 ± 0.7cd	177.9 ± 0.8c	34.6 ± 0.5cd
Ri + Bs + Tv + Fo	42.3 ± 1.4b	122.7 ± 0.8cd	177.4 ± 1.2c	34.6 ± 1.2cd
Fm + Pf + Th + Fo	42.5 ± 1.0b	125.4 ± 0.8bc	181.7 ± 0.9b	36.4 ± 0.8c
Fm + Pf + Tv + Fo	42.8 ± 1.1b	127.9 ± 1.2b	181.0 ± 0.9b	38.5 ± 0.4b

*Fm, Funneliformis mosseae; Ri, Rhizophagus intraradices; Bs, Bacillus subtilis PS1-3; Pf, Pseudomonas fluorescens PS2-6; Tv, Trichoderma virens 140012; Th, Trichoderma harzianum 140015; Fo, Fusarium oxysporum f. sp. radicis-lycopersici. Mean data ± SE of 54 treatments are presented. For each column, data followed by the same letter are not significantly different at p < 0.05 (LSD test).*

### Effects of Symbiotic Microbes on Physiological Metabolism of Tomato Plants

Using non-target LC-MS, metabolome analysis was conducted on the shoots (S) and roots (R) of control (1), Ri + Pf + Th (22), Fo (28), and Ri + Pf + Th + Fo (49) treatments. Principal component analysis (PCA) and partial least squares discriminant analysis were used to analyze the detected MS data to determine the final difference in charge-to-mass ratio, which was then compared with the KEGG metabolite database, and the difference in metabolite content and species among treatments was identified.

### Overview of Metabolite Profiles Using PCA and Hierarchical Cluster Analysis

**FIGURE 1:**
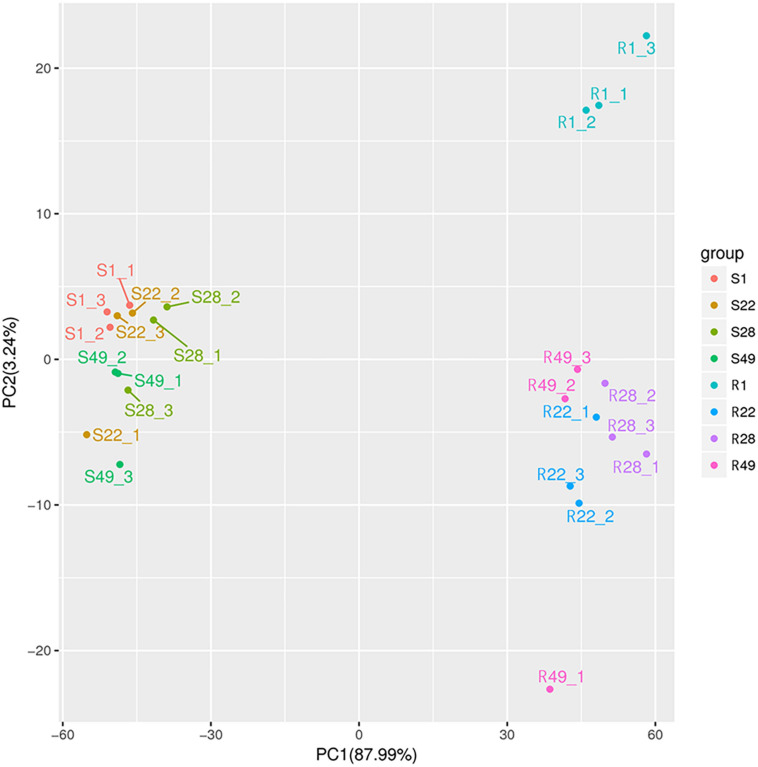
Principal component analysis of tomato shoot (S1, S22, S28, and S49) and root (R1, R22, R28, and R49). 1, Control; 22, Ri + Pf + Th; 28, Fo; 49, Ri + Pf + Th + Fo.

**FIGURE 2:**
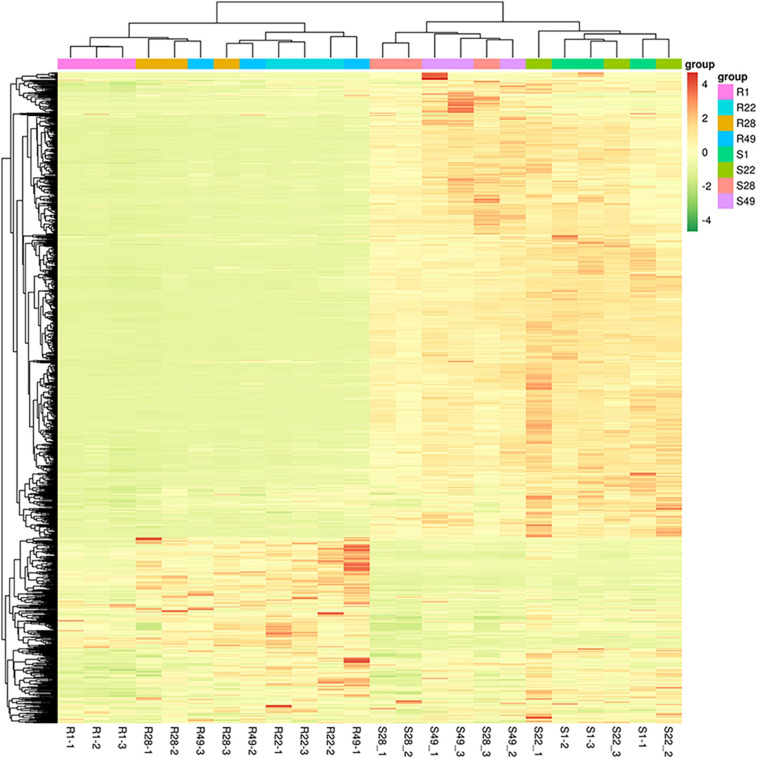
Hierarchical cluster analysis of differential metabolites in tomato shoots (S1, S22, S28, and S49) or roots (R1, R22, R28, and R49). 1, Control; 22, Ri + Pf + Th; 28, Fo; 49, Ri + Pf + Th + Fo.

Principal component analysis was performed to reduce the dimensionality of the data and visualize the relationships among the 24 samples. The first principal component (PC1) explained 87.99% of the total variation and the second principal component (PC2) explained 3.24% of the variation across the data set (Figure 1). The responses of the aboveground and underground organs to the different inoculation treatments were well separated by PC1. Regardless of the control group and the treatment group, no significant difference in the metabolites of the aboveground or underground organs was detected, whereas the metabolites differed between the aboveground and underground organs, indicating that aboveground and belowground metabolism of the plant differed significantly. In addition, the biological replicates were projected closely in multidimensional space, which indicated that the replicates showed a strong correlation. Cluster heat map analysis was conducted for 24 samples, of which all samples were clustered into four main groups and the samples from the same organ were clustered together (Figure 2). A higher number of metabolites were up-regulated in the shoot, whereas a greater number of metabolites were down-regulated in the root of tomato plants (Figure 2). Thus, significant differences in metabolism between aboveground and underground organs of the plant were demonstrated. Correlation analysis among the 24 samples showed that each treatment showed high repeatability. Collectively, these results indicated that the data were reliable and the experiment was meaningful.

### Differential Metabolite Analysis

**FIGURE 3:**
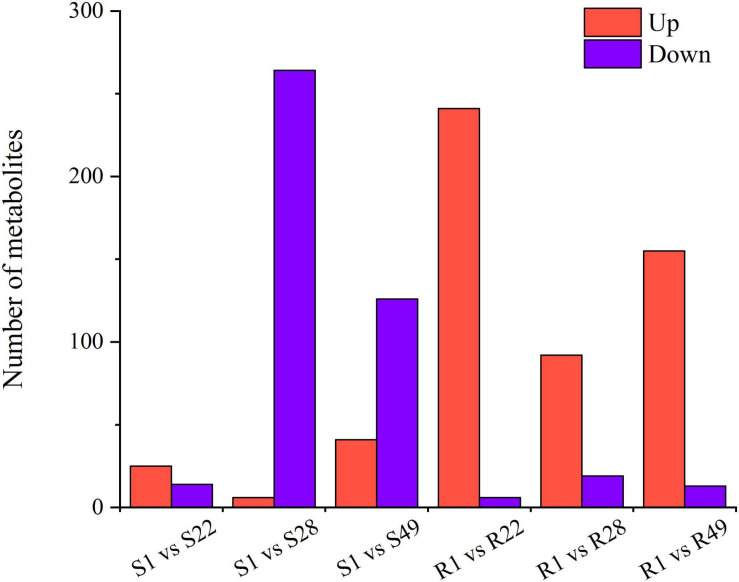
Differential metabolites of S1 vs. S22, S1 vs. S28, S1 vs. S49, R1 vs. R22, R1 vs. R28, and R1 vs. R49. 1, Control; 22, Ri + Pf + Th; 28, Fo; 49, Ri + Pf + Th + Fo.

A total of 1,266 differential metabolites were detected in the metabolome analysis. With regard to the aboveground organs of tomato, compared with the S1 group, the number of up-regulated metabolites in the S22 treatment group exceeded the number of down-regulated metabolites, whereas the number of down-regulated metabolites in the S28 treatment group was significantly increased and was 44 times higher than that of up-regulated metabolites. The number of metabolites down-regulated in the S49 treatment group was more than the number that were up-regulated. However, after inoculation with Ri + Pf + Th, in tomato shoots the number of down-regulated metabolites decreased and the number of up-regulated metabolites increased (Figure 3 and Supplementary Figure 2).

With regard to the roots of tomato, the number of up-regulated metabolites in the R22, R28, and R49 treatment groups was significantly higher than that in the R1 group, and the number of up-regulated metabolites in the R22 treatment group was 40 times higher than the number of down-regulated metabolites. However, compared with the plants inoculated with Fo, the number of up-regulated metabolites increased by 63 and the number of down-regulated metabolites decreased by 6 in tomato inoculated with Ri + Pf + Th (Figure 3 and Supplementary Figure 2).

Thus, it was noted that symbiotic microorganisms or pathogens mainly affected the amount of down-regulated metabolites in the shoots of tomato and participated in the metabolic regulation of aboveground organs. In contrast, in the roots of tomato plants, the number of up-regulated metabolites was more strongly affected by symbiotic microorganisms or pathogens. In addition, significant differences in secondary metabolism between the shoots and roots of tomato plants were observed (Figure 3 and Supplementary Figure 2).

We screened the differential metabolites using the criteria variable importance plot >1 and *p*-value < 0.05 based on a partial least squares-discriminant analysis model of the shoots and roots from different treatments (Supplementary Figure 1).

For the shoot of tomato, it was observed that nine of the top 20 differential metabolites were detected in the Ri + Pf + Th treatment compared with the Fo treatment, comprising four up-regulated and five down-regulated metabolites. Inoculation with Ri + Pf + Th promoted the synthesis of acetylspiramycin and 6-hydroxypentadecanedioic acid, but inhibited the production of glucocaffeic acid, vanillic acid-4-*O*-glucuronide, and 6-caffeoylsucrose (Table 6). In addition, compared with the Fo treatment, 13 of the top 20 differential metabolites were detected in the shoot in the Ri + Pf + Th + Fo group, comprising four up-regulated and nine down-regulated metabolites. Inoculation with Ri + Pf + Th promoted the synthesis of fucosyllactose, zidovudine, and dihydrovaltrate, but inhibited the production of picolinic acid, curcumin, indoleacetic acid, buchanine, and other metabolites (Table 7).

**TABLE 6:** Nine differential metabolites detected in the Ri + Pf + Th treatment compared with Fo treatment from tomato shoots.

Compounds	Description	Metabolic level	Fold change
Meta_8261	Unknown	Up	2.44
Meta_9662	Acetylspiramycin	Up	1.62
Meta_9336	PS [20:3(8Z,11Z,14Z)/18:1(9Z)]	Up	1.48
Meta_2302	6-Hydroxypentadecanedioic acid	Up	1.37
Meta_3569	Glucocaffeic acid	Down	−1.62
Meta_3474	Vanillic acid-4-*O*-glucuronide	Down	−1.7
Meta_6199	6-Caffeoylsucrose; clindamycin phosphate	Down	−1.71
Meta_5782	6-Caffeoylsucrose; clindamycin phosphate	Down	−1.79
Meta_5869	6-Caffeoylsucrose; clindamycin phosphate	Down	−2.18

**TABLE 7:** Thirteen differential metabolites detected in the Ri + Pf + Th + Fo treatment compared with Fo treatment from tomato shoots.

Compounds	Description	Metabolic level	Fold change
Meta_5617	Fucosyllactose	Up	3.05
Meta_5869	6-Caffeoylsucrose; clindamycin phosphate	Up	2.97
Meta_6354	Zidovudine	Up	2.75
Meta_4498	Dihydrovaltrate	Up	2.7
Meta_1432	Methyl 3-carbazolecarboxylate	Down	−1.2
Meta_352	Picolinic acid	Down	−1.25
Meta_3652	Curcumin	Down	−1.27
Meta_735	Indoleacetic acid	Down	−1.29
Meta_2237	Buchanine	Down	−1.31
Meta_2915	Polixetonium chloride	Down	−1.66
Meta_8940	Aquifoliunine EIII	Down	−1.72
Meta_6922	Unknown	Down	−1.89
Meta_6762	LysoPC (22:1(13Z))	Down	−2.31

Compared with inoculation with Fo, 10 of the top 20 differential metabolites were detected in the root after Ri + Pf + Th + Fo inoculation. The metabolites lactosamine, lucuminic acid, cyclomorusin, primidone, icaceine, and 3β,15α-diacetoxylanosta-8,24-dien-26-oic acid were up-regulated, whereas argiopinin I, 16-hydroxyhexadecanoic acid, pantoyllactone glucoside, and sinapoylspermine were down-regulated in the Ri + Pf + Th treatment (Table 8). In addition, compared with the Fo treatment, 11 of the top 20 differential metabolites were detected in the Ri + Pf + Th + Fo treatment. The metabolites lucuminic acid, cyclomorusin, and lactosamine were highly accumulated, whereas quercetin 3-arabinoside 7-glucoside, quercetin 3-glucoside 7-xyloside, and 3-hexadecanoyloleanolic acid were down-regulated in Ri + Pf + Th + Fo treatment (Table 9).

**TABLE 8:** Ten differential metabolites detected in the Ri + Pf + Th treatment compared with Fo treatment in tomato roots.

Compounds	Description	Metabolic level	Fold change
Meta_8579	Argiopinin I	Down	−1.11
Meta_2422	16-Hydroxy hexadecanoic acid	Down	−1.22
Meta_3346	Pantoyllactone glucoside	Down	−1.27
Meta_4259	Sinapoylspermine	Down	−1.55
Meta_3155	Lactosamine	Up	4.02
Meta_5127	Lucuminic acid	Up	3.96
Meta_4732	Cyclomorusin	Up	3.83
Meta_1926	Primidone	Up	3.61
Meta_4111	Icaceine	Up	3.59
Meta_8040	3beta,15alpha-Diacetoxylanosta-8,24-dien-26-oic acid	Up	3.39

**TABLE 9:** Eleven differential metabolites detected in the Ri + Pf + Th + Fo treatment compared with Fo treatment in tomato roots.

Compounds	Description	Metabolic level	Fold change
Meta_5127	Lucuminic acid	Up	4.86
Meta_4732	Cyclomorusin	Up	4.59
Meta_3155	Lactosamine	Up	3.9
Meta_8376	Unknown	Down	−0.9
Meta_6922	Unknown	Down	−0.9
Meta_7076	Quercetin 3-arabinoside 7-glucoside	Down	−1.11
Meta_8227	Quercetin 3-glucoside 7-xyloside	Down	−1.12
Meta_7919	3-Hexadecanoyloleanolic acid	Down	−1.12
Meta_8593	Unknown	Down	−1.29
Meta_8671	Unknown	Down	−1.36
Meta_8579	Argiopinin I	Down	−1.49

**FIGURE 4:**
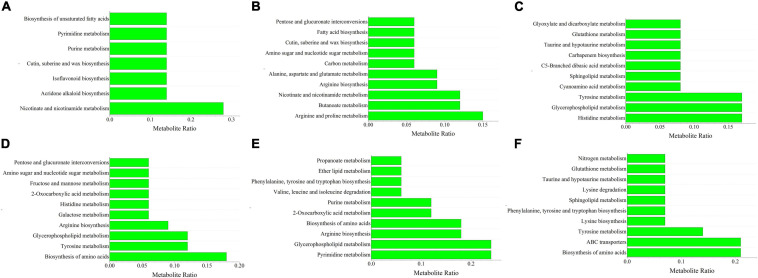
Top 10 metabolites ratio of S1 vs. S22 **(A)**, S1 vs. S28 **(B)**, S1 vs. S49 **(C)**, R1 vs. R22 **(D)**, R1 vs. R28 **(E)**, and R1 vs. R49 **(F)**. 1, Control; 22, Ri + Pf + Th; 28, Fo; 49, Ri + Pf + Th + Fo.

A KEGG enrichment analysis was performed (Supplementary Figure 3). Nicotinate and nicotinamide metabolism pathways were the most abundant pathways in tomato shoots after inoculation with Ri + Pf + Th and constituted 28% of the differential metabolites detected. After single inoculation with Fo, arginine and proline metabolism was most strongly enriched pathway in the shoots and comprised 15% of the metabolites, followed by nicotinate and nicotinamide metabolism and butanoate metabolism (12%). After inoculation with Ri + Pf + Th + Fo, 17% of the total metabolites were mainly enriched in the histidine metabolism, glycerophospholipid metabolism, and tyrosine metabolism pathways in tomato shoots (Figure 4).

Among the top 10 enriched pathways, 18% of the total metabolites were involved in biosynthesis of amino acids in tomato roots inoculated with Ri + Pf + Th, and 12% of the metabolites were enriched with the tyrosine metabolism and glycerophospholipid metabolism pathways. After single inoculation with Fo, 24% of the metabolites in the roots were enriched in the pyrimidine metabolism and glycerophospholipid metabolism pathways, and 18% of the metabolites were enriched in the arginine biosynthesis and amino acid biosynthesis pathways. The amino acid biosynthesis and ABC transporters pathways were the most strongly enriched pathways in tomato roots after inoculation with Ri + Pf + Th + Fo, accounting for 21% of the total metabolites, whereas 14% of the metabolites were involved in the tyrosine metabolism pathway (Figure 4). It was speculated that the amino acid biosynthesis pathway played an important role in promoting root growth and development of tomato, and the ABC transporters pathway may play a pivotal role in resistance to pathogenic bacteria in tomato roots.

## Discussion

Increasing research attention is focused on disease control by symbiotic microbe combinations. Kabdwal et al. (2019) conducted growth promotion and disease control experiments on tomato using *T. harzianum* (Th43), *P. fluorescens* (Pf173), *R. intraradices*, and mancozeb, which was applied in different combinations as a soil application, seedling treatment, or foliar spray. The authors observed that soil treated with Th43 + Pf173 + *R. intraradices* and seedlings treated with Th43 + Pf173 + three applications of mancozeb as a foliar spray were highly effective in reducing plant mortality, promoting plant growth, and increasing yield. A field trial by Kumar et al. (2018) showed that *Glomus mosseae* and *Glomus fasciculatum* + *Trichoderma viride* (TR) + *Azotobacter chroococcum* (AZ) combinations had the strongest effect on litchi growth. In the present study, we observed that the combinations Ri + Bs + Tv and Fm + Pf + Tv had the greatest growth-promoting effects on tomato, whereas the combination Ri + Pf + Th had the greatest biological control effect against Fo. Differing from non-symbiotic microbial biocontrol agents, symbiotic microorganisms were used in the present experiment. These AMF, PGPR, and *Trichoderma* spp. colonized the rhizosphere as well as the root system, thereby forming a mutual symbiosis. *Trichoderma* spp. not only exist widely in the soil but also colonize the surface and internal tissues of plant roots, stems, and leaves. Zhang et al. (2013) observed that *T. harzianum* T-E5 colonized the root system of cucumber in a hydroponic culture system; first, the mycelia covered the root surface, then gradually extended into the cortex of the root, and survived in the epidermis and exodermis of the root for a long period. In the present experiment, both *T. viride* and *T. harzianum* colonized tomato root cortical cells but differed in their colonization ability, especially after inoculation with a pathogenic fungus. The colonization frequency of *T. viride* was higher than that of *T. harzianum*, thus it could be inferred that the colonization ability of the former species was stronger, which may affect the capability to prevent and control disease. The present results also confirmed this point. This finding is of great importance and warrants further systematic investigation. Furthermore, we observed that *Trichoderma* spp. colonized tomato roots together with AMF to form complex symbionts, and played a synergistic role with AMF, or concurrently exerted an ecological effect in collaboration with AMF + PGPR. This is the first observation of a complex symbiont formed by AMF + *Trichoderma*. The present experiment not only confirmed the aforementioned effects of *Trichoderma* but further demonstrated that *Trichoderma* played a synergistic role in combination with AMF or formed a biologically effective collaboration with AMF + PGPR. Therefore, it is of practical importance to further investigate the physiological and ecological functions and mechanism of the AMF + *Trichoderma* + PGPR symbiosis.

Symbiotic microorganisms function by direct synthesis of secondary metabolites, such as phytohormones and antibiotics, regulation of plant-related gene expression, and regulation of the community structure of other organisms. In the process of coevolution of plants and pathogens, complex and diverse defense systems have developed. Among these systems, many metabolites produced by plant metabolic pathways are an important material basis of plant resistance and play an important role in the plant defense system. Using LC-MS technology, Tian et al. (2019) reported that resistant and susceptible tobacco cultivars infected by *Meloidogyne incognita* both showed significant changes in the metabolic pathways associated with disease resistance. The differential metabolites identified included alkaloids, fatty acids, flavonoids, terpenoids, and polyketones. A total of 1,374 proteins and 33 small molecular metabolites in grape leaves were differentially expressed by 1.5 times (*p* < 0.05) after infection by *Botrytis cinerea* for 3 days. Infection by *B. cinerea* had the greatest influence on expression of chloroplast proteins and mainly affected the three signaling pathways of plant disease interaction, plant hormone synthesis, and alkaloid synthesis. In addition, full activation of the disease resistance signaling pathway mediated by salicylic acid was an effective means to resist infection by *B. cinerea* in grape leaves (Fang et al., 2019). The present metabolome analysis provided results similar to the aforementioned findings, with a total of 1,266 differential metabolites detected and a large number of unknown metabolites. The results showed that AMF + PGPR + *Trichoderma* inoculation promoted the synthesis of secondary metabolites, including antibiotics and acids, in tomato leaves, but inhibited the synthesis of certain hydrocarbons. Furthermore, this combination promoted the synthesis of antibiotics, acids, and other hydrocarbons in the roots, but inhibited the secretion of acids, glycosides, and certain nitrogen-containing compounds by the roots. In contrast, AMF + PGPR + *Trichoderma* + Fo co-inoculation promoted the synthesis of secondary metabolites, including saccharides, nucleoside compounds, and hydrocarbons in tomato leaves, but inhibited the synthesis of certain pigments, auxin, and bactericide. Furthermore, the combination promoted the synthesis of glycosides and hydrocarbons in roots, but inhibited secretion of certain glycoside compounds by the roots. The number of metabolites up-regulated and down-regulated in each combination differed significantly, but the specific differences in metabolites and metabolic pathways require further study. The results indicated that a compound microbial agent generated by a certain combination of multiple symbiotic microorganisms may be more effective than a single microbial agent. Therefore, in future research and development of green technologies for prevention and control of TFCRR, the screening and evaluation of compound symbiotic bacteria should be a focus.

The present results may help to develop microbial strain resources and to use the advantages of multiple microbial strains. This will contribute to the development of novel microbial pesticides and biofertilizers for effective pest control. It will be helpful to determine whether there are antagonistic effects between the symbiotic microbes, in addition to their growth-promoting effect, when inoculated in combinations. The present study focused on biological disease prevention as a measure to provide a theoretical basis to eliminate the environmental damage caused by abuse of chemical fertilizers and pesticides. The findings also provide guidance for further research and development of green disease prevention and control technologies and promote agricultural sustainability and economic development.

Given the temporal and spatial limitations of the present experiment, environmental factors such as light, moisture, temperature, and insect pests were not considered but may impact on the biocontrol effectiveness of combinations of AMF, PGPR, and *Trichoderma* spp. In addition, the metabolome analysis in this experiment has certain shortcomings, such as less processing number. Therefore, single- and dual-inoculation treatments should be analyzed further in future experiments to obtain more complete metabolite profiles and clarify the metabolic pathways that promote tomato growth and resistance to TFCRR. Simultaneous analysis of gene expression and the transcriptome could be conducted. Optimization of the promotive effect of different types of symbiotic microbes and minimization of antagonistic interactions, thereby producing more effective biocontrol bacterial agents, will further contribute to development of green technologies for disease prevention and control.

## Conclusion

Applications of symbiotic microbes, alone or in combinations, influence leaf photosynthesis, defense enzyme activities, disease occurrence, growth and development, and yield of tomato. Combinations of AMF, PGPR, and *Trichoderma*, such as *R. intraradices* + *P. fluorescens* + *T. harzianum*, have strong biocontrol effects on TFCRR. Therefore, this symbiotic microbe combination shows potential for development as an effective biocontrol agent.

## Data Availability Statement

The original contributions generated for this study are included in the article/Supplementary Material, further inquiries can be directed to the corresponding author.

## Author Contributions

XC and ML conceived, designed, carried out the experiments, and collected and analyzed the data. HZ and CL prepared the experimental materials. XC wrote the first draft of the manuscript. RL and ML revised the manuscript. All authors approved the final version of the article for publication.

## Conflict of Interest

The authors declare that the research was conducted in the absence of any commercial or financial relationships that could be construed as a potential conflict of interest.

## Supplementary Material

Supplementary Figure 1 Differential multiple histogram. Top 20 differential metabolites of S1 vs. S22 **(A)**, S1 vs. S28 **(B)**, S1 vs. S49 **(C)**, R1 vs. R22 **(D)**, R1 vs. R28 **(E)**, and R1 vs. R49 **(F)**. 1, Control; 22, Ri + Pf + Th; 28, Fo; 49, Ri + Pf + Th + Fo.

Supplementary Figure 2 Volcanic map of differential metabolites. Differential metabolite volcanic maps of S1 vs. S22 **(A)**, S1 vs. S28 **(B)**, S1 vs. S49 **(C)**, R1 vs. R22 **(D)**, R1 vs. R28 **(E)**, and R1 vs. R49 **(F)**. 1, Control; 22, Ri + Pf + Th; 28, Fo; 49, Ri + Pf + Th + Fo.

Supplementary Figure 3 The most enriched KEGG pathway of differential metabolites, as analyzed via pairwise comparisons between different treatments. Different metabolites KEGG enrichment maps of S1 vs. S22 **(A)**, S1 vs. S28 **(B)**, S1 vs. S49 **(C)**, R1 vs. R22 **(D)**, R1 vs. R28 **(E)**, and R1 vs. R49 **(F)**. 1, Control; 22, Ri + Pf + Th; 28, Fo; 49, Ri + Pf + Th + Fo.

Supplementary Table 1 The list of treatments.

Supplementary Table 2 Metabolites_full_table (NEG).

Supplementary Table 3 Metabolites_full_table (POS).

## References

[B1] Atalla S. M. M. Abdel-Kader M. M. El-Gamal N. G. El-Mougy N. S. (2020). Using maize wastes, fermented by co-cultures of *Trichoderma harzianum* and *Pseudomonas fluorescens*, as grain dressing against m maize diseases under field conditions. *Egypt. J. Biol. Pest Control* 30:37. 10.1186/s41938-020-00236-x

[B2] Bashan Y. Holguin G. Lifshitz R. (1993). “Isolation and characterization of plant growth-promoting rhizobacteria,” in *Methods in Plant Molecular Biology and Biotechnology*, eds Glick B. R. Thompson J. E. (Boca Raton, FL: CRC Press).

[B3] Biermann B. Linderman R. G. (1981). Quantifying vercular-arbuscular mycorrhizas: a proposed method towards standardization. *New Phytol.* 87 63–67. 10.1111/j.1469-8137.1981.tb01690.x

[B4] Cao H. Li X. Wang X. Bai H. Mu W. Liu F. (2018). Control efficacy of pyraclostrobin and triazole fungicides against tomato crown and root rot. *Sci. Agric. Sin.* 51 4065–4075. 10.3864/j.issn.0578-1752.2018.21.006

[B5] Colak A. Bicici M. (2013). Integrated disease management of *Fusarium* crown and root rot of greenhouse-grown tomato in eastern Mediterranean region of turkey. *Tarim Bilim. Derg.* 19 89–100.

[B6] Dearth S. P. Castro H. F. Venice F. Tague E. D. Novero M. Bonfante P. (2018). Metabolome changes are induced in the arbuscular mycorrhizal fungus *Gigaspora margarita* by germination and by its bacterial endosymbiont. *Mycorrhiza* 28 421–433. 10.1007/s00572-018-0838-8 29860608

[B7] Fang X. He Y. Xi X. Zha Q. Zhang L. Jiang A. (2019). Multi-omics reveals the resistance mechanism of grape leaves in response to *Botrytis cinerea*. *J. Zhejiang Univ. (Agric. Life Sci.)* 45 306–316. 10.3785/j.issn.1008-9209.2018.11.121

[B8] Hibar K. Daami-Remadi M. Ayed F. Mahjoub M. E. (2007). *Fusarium* crown and root rot of tomato and its chemical control. *Int. J. Agric. Res.* 2 687–695. 10.3923/ijar.2007.687.695

[B9] Hibar K. Daami-Remadi M. Hamada W. El-Mahjoub M. (2006). Bio-fungicides as an alternative for tomato *Fusarium* crown and root rot control. *Tunis. J. Plant Prot.* 1 19–29.

[B10] Hill E. M. Robinson L. A. Abdul-Sada A. Vanbergen A. J. Hodge A. Hartley S. E. (2018). Arbuscular mycorrhizal fungi and plant chemical defence: effects of colonisation on aboveground and belowground metabolomes. *J. Chem. Ecol.* 44 198–208. 10.1007/s10886-017-0921-1 29392532 PMC5843688

[B11] Kabdwal B. C. Sharma R. Tewari R. Tewari A. K. Singh R. P. Dandona J. K. (2019). Field efficacy of different combinations of *Trichoderma harzianum*, *Pseudomonas fluorescens*, and arbuscular mycorrhiza fungus against the major diseases of tomato in Uttarakhand (India). *Egypt. J. Biol. Pest Control* 29:1. 10.1186/s41938-018-0103-7

[B12] Kucharek T. Jones J. P. Hopkins D. Strandberg I. (2000). *Some disease of vegetables and agronomic crops caused by Fusarium in Florida*. Circular 1025, Florida Cooperative Extension Service. Gainesville, FL: Institute of Food and Agricultural Sciences, University of Florida.

[B13] Kumar V. Anal A. K. D. Nath V. (2018). Growth response of litchi to arbuscular mycorrhizal co-inoculation with *Trichoderma viride*, *Azotobacter chroococcum* and *Bacillus megatarium*. *Indian Phytopath.* 71 65–74. 10.1007/s42360-018-0010-6

[B14] Li J. Sun Y. Zhao T. Jiang J. Xu X. (2018). Separation identification and biological characteristics of pathogen causing *Fusarium* crown and root rot of tomato. *J. Northeast Agric. Univ.* 49 22–30.

[B15] Liu B. Wang Y. Hu J. Li J. Yang H. (2019). Biocontrol effect evaluation of combined of *Trichoderma harzianum* and *Bacillus cereus* against root-knot nematode *Meloidogyne* ssp. on tomatoes. *Shandong Sci.* 32 71–75. 10.3976/j.issn.1002-4026.2019.05.008

[B16] Liu D. Li M. Sun W. Liu R. (2016). Selection of combinations of arbuscular mycorrhizal fungi and plant growth-promoting rhizobacteria against cucumber *Fusarium* wilt disease. *Acta Phytopathol. Sin.* 46 821–832. 10.13926/j.cnki.apps.2016.06.012

[B17] Liu D. Li M. Sun W. Liu R. (2017). Mechanism of increasing resistance of cucumber plants to *Fusarium* wilt disease by combined inoculation with arbuscular mycorrhizal fungi and plant growth-promo- ting rhizobacteria. *Acta Phytopathol. Sin.* 47 832–841.

[B18] Liu R. Luo X. (1994). A new method to quantify the inoculum potential of arbuscular mycorrhizal fungi. *New Phytol.* 128 89–92. 10.1111/j.1469-8137.1994.tb03990.x 33874524

[B19] Liu R. Wang L. (2018). *Biological Symbiotics.* Beijing: Science press.

[B20] Lu C. C. Guo N. Yang C. Sun H. B. Cai B. Y. (2020). Transcriptome and metabolite profiling reveals the effects of *Funneliformis mosseae* on the roots of continuously cropped soybeans. *BMC Plant Biol.* 20:479. 10.1186/s12870-020-02647-2 33087042 PMC7579952

[B21] Mendes J. B. S. da Costa Neton V. P. de Sousa C. D. A. de Carvalho Filho M. R. Rodrigues A. C. Bonifacio A. (2020). *Trichoderma* and bradyrhizobia act synergistically and enhance the growth rate, biomass and photosynthetic pigments of cowpea (*Vigna unguiculata*) grown in controlled conditions. *Symbiosis* 80 133–143. 10.1007/s13199-019-00662-y

[B22] Mikiciuk G. Sas-Paszt L. Mikiciuk M. Derkowska E. Trzciński P. Głuszek S. (2019). Mycorrhizal frequency, physiological parameters, and yield of strawberry plants inoculated with endomycorrhizal fungi and rhizosphere bacteria. *Mycorrhiza* 29 489–501. 10.1007/s00572-019-00905-2 31264099

[B23] Murugesan S. Vijayakumar R. Panneerselvam A. (2011). Antifungal activity of medicinal plants against plant pathogenic fungus *Fusarium oxysporum*. *J. Pharm. Res.* 4 843–844.

[B24] Nanjundappa A. Bagyaraj D. J. Saxena A. K. Kumar M. Chakdar H. (2019). Interaction between arbuscular mycorrhizal fungi and *Bacillus* spp. in soil enhancing growth of crop plants. *Fungal Biol. Biotechnol.* 6:23. 10.1186/s40694-019-0086-5 31798924 PMC6882151

[B25] Pan X. Chang R. Mu K. Chen Q. (2020). Inhibition effects of *Trichoderma harzianum* VT9-3r and Bacillus subtilis VT4-1x on three potato pathogens. *J. China Agric. Univ.* 25 72–81.

[B26] Rana K. L. Kour D. Kaur T. Devi R. Yadav A. N. Yadav N. (2020). Endophytic microbes: biodiversity, plant growth-promoting mechanisms and potential applications for agricultural sustainability. *Antonie van Leeuwenhoek* 113 1075–1107. 10.1007/s10482-020-01429-y 32488494

[B27] Tan S. Sun W. Liu R. (2015). Combination of *Glomus* spp. and *Bacillus* sp. M3-4 promotes plant resistance to bacterial wilt in potato. *Acta Phytopathol. Sin.* 45 661–669.

[B28] Thorpe H. J. Jarvis W. R. (1981). Grafted tomatoes escape *Fusarium* foot and root rot. *Can. J. Plant Sci.* 61 1027–1028. 10.4141/cjps81-157

[B29] Tian P. Li X. He W. Duan D. Xu S. Yang T. (2019). Metabonomics analysis of resistant susceptible tobacco varieties before and after infection by *Meloidogyne incognita*. *Acta Tabacaria Sin.* 25 81–92. 10.16472/j.chinatobacco.2018.261

[B30] Wang J. Ren P. Zhang F. Hong B. Chang Q. Liu C. (2019). “Isolation and identification of pathogen of tomato Fusarium crown and root rot in greenhouse,” in *Proceedings of the 2019 Annual Meeting of the Society for Plant Protection China Society of Plant Protection*, 81–86.

[B31] Wang X. Chen M. Yang G. Li X. Li P. Chen M. (2014). Effect of *Glomus versiforme* and *Trichodema harzianum* on growth and quality of *Salvia miltiorrhiza*. *China J. Chin. Mater. Med.* 39 1574–1578. 10.4268/cjcmm20140906 25095363

[B32] Wang X. K. (2006). *Principles and Techniques of Plant Physiological Biochemical Experimental.* Beijing: Higher Education Press.

[B33] Yang C. X. Zhao W. N. Wang Y. N. Zhang L. Huang W. C. Lin J. X. (2020). Metabolomics analysis reveals the alkali tolerance mechanism in *Puccinellia tenuiflora* Plants inoculated with arbuscular mycorrhizal fungi. *Microorganisms* 8:327. 10.3390/microorganisms8030327 32110985 PMC7142761

[B34] Zhang F. Zhu Z. Wang B. Wang P. (2013). Optimization of *Trichoderma harzianum* T-E5 biomass and determining the degradation sequence of biopolymers by FTIR in solid-state fermentation. *Ind. Crops Prod.* 49 619–627. 10.1016/j.indcrop.2013.05.037

[B35] Zhao B. He S. (2002). *Microbiology Experiments.* Beijing: Science Press.

