# Supplementary figures and images for: Effects and Mechanisms of Symbiotic Microbial Combination Agents to Control Tomato Fusarium Crown and Root Rot Disease
Source: Front Microbiol. 2021 Jun 17;12:629793. doi: 10.3389/fmicb.2021.629793 (PMC8245789; doi:10.3389/fmicb.2021.629793)

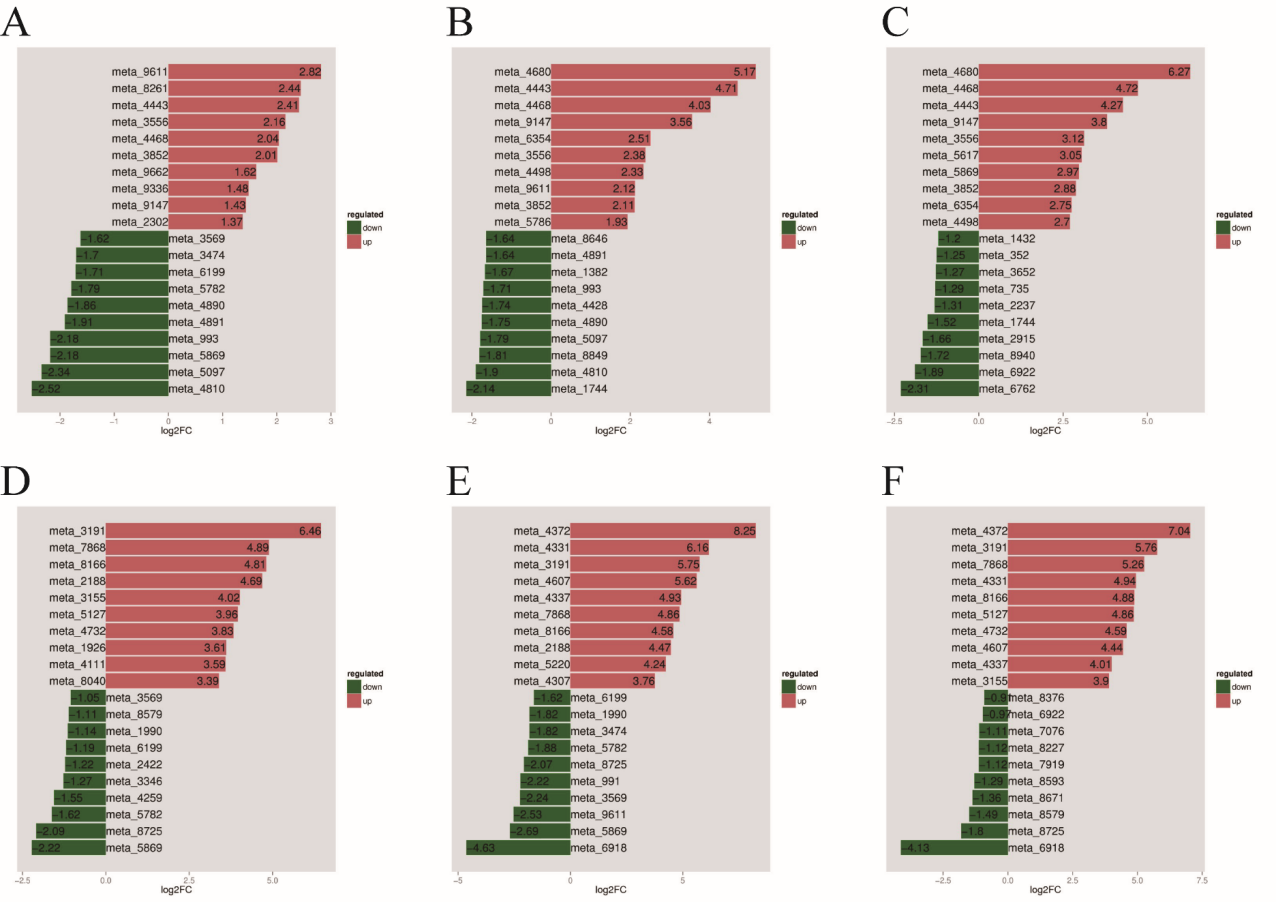

Supplement: Supplementary Figure 1 — Differential multiple histogram. Top 20 differential metabolites of S1 vs. S22 (A), S1 vs. S28 (B), S1 vs. S49 (C), R1 vs. R22 (D), R1 vs. R28 (E), and R1 vs. R49 (F). 1, Control; 22, Ri + Pf + Th; 28, Fo; 49, Ri + Pf + Th + Fo. [file Image_1.TIF]

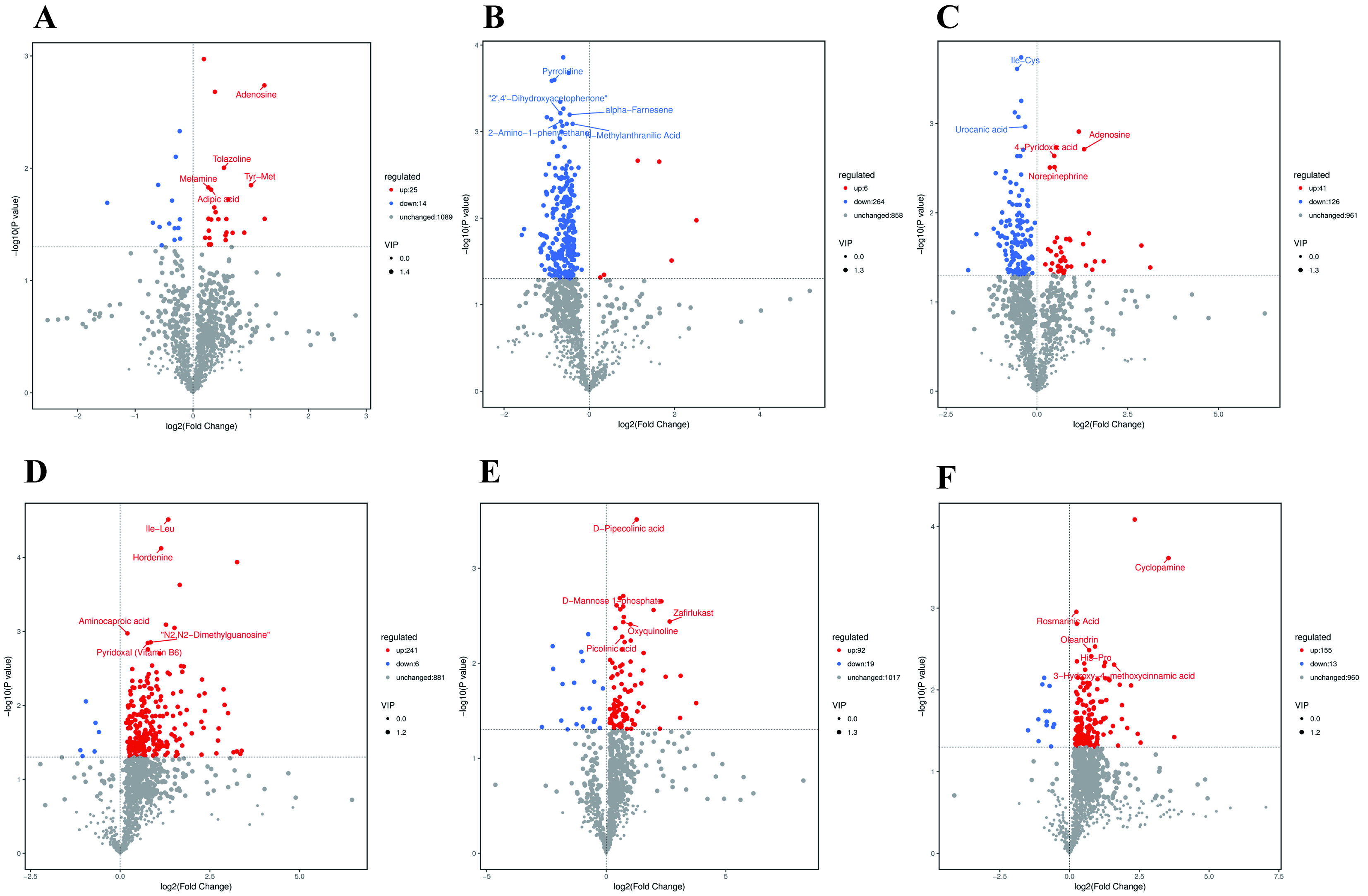

Supplement: Supplementary Figure 2 — Volcanic map of differential metabolites. Differential metabolite volcanic maps of S1 vs. S22 (A), S1 vs. S28 (B), S1 vs. S49 (C), R1 vs. R22 (D), R1 vs. R28 (E), and R1 vs. R49 (F). 1, Control; 22, Ri + Pf + Th; 28, Fo; 49, Ri + Pf + Th + Fo. [file Image_2.TIF]

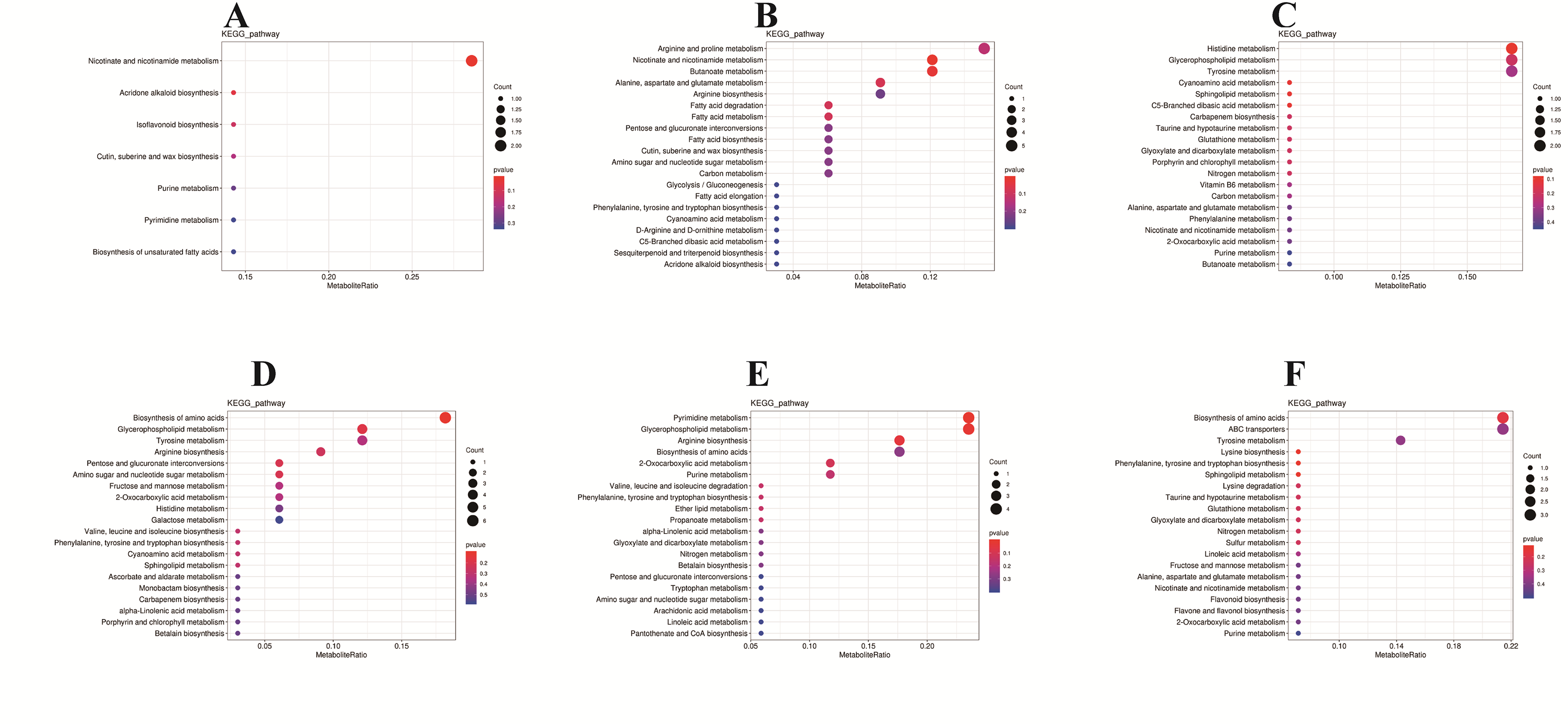

Supplement: Supplementary Figure 3 — The most enriched KEGG pathway of differential metabolites, as analyzed via pairwise comparisons between different treatments. Different metabolites KEGG enrichment maps of S1 vs. S22 (A), S1 vs. S28 (B), S1 vs. S49 (C), R1 vs. R22 (D), R1 vs. R28 (E), and R1 vs. R49 (F). 1, Control; 22, Ri + Pf + Th; 28, Fo; 49, Ri + Pf + Th + Fo. [file Image_3.TIF]
